# Minimal Size of Cell Assemblies Coordinated by Gamma Oscillations

**DOI:** 10.1371/journal.pcbi.1002362

**Published:** 2012-02-09

**Authors:** Christoph Börgers, Giovanni Talei Franzesi, Fiona E. N. LeBeau, Edward S. Boyden, Nancy J. Kopell

**Affiliations:** 1 Department of Mathematics, Tufts University, Medford, Massachusetts, United States of America; 2 MIT Media Lab, Massachusetts Institute of Technology, Cambridge, Massachusetts, United States of America; 3 Institute of Neuroscience, The Medical School, Newcastle University, Newcastle Upon Tyne, United Kingdom; 4 Department of Mathematics and Center for Biodynamics, Boston University, Boston, Massachusetts, United States of America; Indiana University, United States of America

## Abstract

In networks of excitatory and inhibitory neurons with mutual synaptic coupling, specific drive to sub-ensembles of cells often leads to gamma-frequency (25–100 Hz) oscillations. When the number of driven cells is too small, however, the synaptic interactions may not be strong or homogeneous enough to support the mechanism underlying the rhythm. Using a combination of computational simulation and mathematical analysis, we study the breakdown of gamma rhythms as the driven ensembles become too small, or the synaptic interactions become too weak and heterogeneous. Heterogeneities in drives or synaptic strengths play an important role in the breakdown of the rhythms; nonetheless, we find that the analysis of homogeneous networks yields insight into the breakdown of rhythms in heterogeneous networks. In particular, if parameter values are such that in a homogeneous network, it takes several gamma cycles to converge to synchrony, then in a similar, but realistically heterogeneous network, synchrony breaks down altogether. This leads to the surprising conclusion that in a network with realistic heterogeneity, gamma rhythms based on the interaction of excitatory and inhibitory cell populations must arise either rapidly, or not at all. For given synaptic strengths and heterogeneities, there is a (soft) lower bound on the possible number of cells in an ensemble oscillating at gamma frequency, based simply on the requirement that synaptic interactions between the two cell populations be strong enough. This observation suggests explanations for recent experimental results concerning the modulation of gamma oscillations in macaque primary visual cortex by varying spatial stimulus size or attention level, and for our own experimental results, reported here, concerning the optogenetic modulation of gamma oscillations in kainate-activated hippocampal slices. We make specific predictions about the behavior of pyramidal cells and fast-spiking interneurons in these experiments.

## Author Summary

Gamma-frequency (25–100 Hz) oscillations in the brain often arise as a result of an interaction between excitatory and inhibitory cell populations. For this mechanism to work, the interaction must be sufficiently strong, and connectivity and external drives to participating neurons must be sufficiently homogeneous. As the interactions become weaker, either because the neuronal ensembles become smaller or because synapses weaken, the rhythms deteriorate, and eventually break down. This fact, by itself, is not surprising, but details of how the breakdown occurs are subtle. In particular, our analysis leads to the conclusion that in realistically heterogeneous networks, gamma rhythms must arise quickly, within a small number of oscillation periods, if they arise at all. Our findings suggest explanations for recent experimental findings concerning the minimal spatial extent of stimuli eliciting gamma oscillations in the primary visual cortex, the modulation of gamma oscillations in the primary visual cortex by attention, as well as our own experimental results, reported here, concerning the minimal light intensity below which optogenetic drive to pyramidal cells in a kainate-activated hippocampal slice results in disruption of an ongoing gamma oscillation. Our analysis leads to experimentally testable predictions about the behavior of the excitatory and inhibitory cells in these experiments.

## Introduction

Mechanisms underlying the formation of gamma-frequency (25–100 Hz) rhythms in networks of excitatory and inhibitory neurons (E- and I-cells) have been investigated extensively [1]–[11]. However, mechanisms underlying the loss of rhythmicity, as parameters change, have been given less attention. Here we consider the loss of gamma rhythmicity as the number of participating cells decreases. We focus on gamma rhythms resulting from the synaptic interaction of E- and I-cells, thinking of pyramidal cells and fast-spiking interneurons interacting via AMPA- and 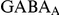-receptor-mediated synapses. The E-cells spike intrinsically, driving and synchronizing the I-cells, which in turn gate and synchronize the E-cells. Rhythms of this kind are called PING (Pyramidal-Interneuronal Network Gamma) rhythms [10], [11]. We distinguish between “strong PING” and “weak PING”. In strong PING, there is strong tonic (i.e., temporally constant) drive to some or all E-cells, and those E-cells that participate at all typically participate on every population cycle. In weak PING, drive to the E-cells is stochastic, and typically each individual E-cell participates only on a fraction of population cycles [12]. We think of weak PING as a reduced model of the kainate-induced persistent gamma rhythm in slice [13]–[15]. Of course, real gamma oscillations might also be a mixture of “strong” and “weak” PING, with a stochastically fluctuating drive added to largely tonic baseline excitation. In most of the simulations of this paper, we omit any stochastic drive for simplicity, and assume that some or all E-cells receive strong constant drive. When the driven ensemble is large and synaptic interactions are strong, a strong PING rhythm will often arise in the driven ensemble [12], [16], [17]. We refer to an ensemble of cells of this sort, firing in synchrony at gamma frequency, as a “cell assembly”.

The breakdown of gamma rhythms, as the number of tonically driven cells is decreased, is the combined effect of weak synaptic interactions and heterogeneity. Therefore the modeling part of the Results section of this paper begins with a study of how strong PING rhythms break down as synapses are weakened in heterogeneous E/I-networks of fixed size, assuming that all E-cells are driven. Using a combination of numerical simulations and mathematical analysis, we show that the breakdown of the rhythm can often be understood well by studying highly reduced, homogeneous networks. In a realistically heterogeneous network, the rhythm breaks down when the E-to-I-synapses become so weak that a single excitatory spike volley is no longer sufficient to prompt a response from the I-cells, or when the I-to-E-synapses become so weak that in a homogeneous network, convergence to tight synchrony would take several gamma periods. A surprising conclusion of our analysis is that a slow, graduate slide into PING, which is possible in a homogeneous network, is not possible in a realistically heterogeneous network; the PING rhythm is either established rapidly, or not at all.

We then apply this analysis to understand how the rhythm breaks down as the number of driven cells is reduced. We conclude that for given synaptic strengths and heterogeneities, there is a (soft) lower bound on the possible size of cell assemblies.

As explained in detail in the Discussion, our modeling results suggest possible theoretical explanations for several recent experimental findings: (1) Gieselmann *et al.* [18], observed no gamma rhythm in primary visual cortex when the spatial extent of the driving stimulus was too small. (2) Chalk *et al.* [19] found that attention can weaken gamma oscillations in primary visual cortex. (3) In this paper, we report that in kainate-bathed hippocampal slices, strong optogenetic drive to the pyramidal cells elicits fast oscillations, whereas weak drive not only fails to elicit fast oscillations, but also abolishes the slower kainate-induced background gamma oscillations [14], [15]. Our results lead to specific predictions concerning the behavior of pyramidal cells and fast-spiking interneurons in these experiments.

## Methods

### Experimental methods

Lentivirus carrying the light-activated cation channel ChIEF [20], an enhanced-performance version of channelrhodopsin-2 [21], [22], under the control of the CaMKII promoter was injected in the CA3 region of C57BL/6 mice. After 3–5 weeks, the animals were sacrificed, and 450 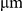-thick horizontal hippocampal slices were cut. Bath application of 400 nM of the glutamatergic agonist kainic acid (Cayman Chemicals) induced 25–50 Hz oscillations in the local field potential (LFP), as recorded in the CA3 stratum radiatum. Light pulses were delivered via a DG-4 optical switch with a 300 W xenon lamp (Sutter Instruments) and GFP filter set (Chroma). For further details on the experimental methods, see Text S1, ection A.

### Computational models

#### Model networks

We describe only the most important features of our network models in this section; for details, see Text S1, ection B. In our model networks, the E-cells are reduced Traub-Miles neurons [17], and the I-cells Wang-Buzsáki neurons [23]. Our models include E-to-I, I-to-E, and I-to-I-synapses. We omit E-to-E-synapses throughout most of this paper. To first approximation, such synapses can be thought of as adding excitation to the E-cells, akin to raising external drive to the E-cells; thus they raise the frequency of the PING rhythm, but do not alter the network behavior qualitatively. For numerical experiments concerning the effects of E-to-E-synapses, see Text S1, Section C.

We begin with networks without any spatial structure, similar to those of Fig. 1 of Ref. [4]. Connectivity is sparse and random. In most of the simulations of this paper, external drives are constant in time but heterogeneous, i.e., inputs to different cells are of different strengths. In some simulations, we drive the E-cells with independent random sequences of excitatory synaptic input pulses, arriving on Poisson schedules [12], [16]. We also consider model networks with spatial structure, assigning to each neuron a random location in the disk of radius 1 centered at the origin in the 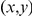-plane. (Distance is non-dimensionalized here.) In such networks, we let the probability (not the strength) of a synaptic connection between two neurons decay exponentially with distance between the neurons.

#### Notation

Synaptic strengths are of paramount importance in this paper. We therefore introduce our notation for these quantities here. (Other notational conventions are introduced in Results as needed.) We denote by 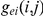 the maximal conductance density associated with the synaptic input to the 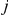-th I-cell from the 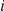-th E-cell, by 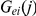 the sum of 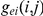 over all 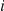, i.e., the total excitatory conductance density impinging upon the 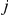-th I-cell, and by 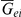 the average of 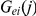 over all I-cells. Quantities 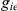, 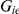, 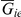, 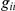, 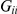, 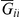, 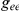, 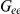, and 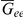 are defined similarly. Thus the small letter 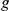 always denotes the conductance density associated with the synaptic interaction between two neurons, whereas the capital 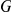 indicates summation over presynaptic cells, and a bar over 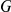 indicates averaging over post-synaptic cells.

#### Quantifying rhythmicity

To study the dependence of rhythmicity on parameters, it is useful to define a quantitative measure of rhythmicity. There are many different ways of doing this, and by necessity the choice is somewhat arbitrary. Since rhythmicity may be relevant to the effectiveness of signals sent by the pyramidal cells in a local network to other parts of the brain, our rhythmicity measure, 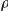, is based on the frequency content of the average, 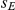, of all gating variables governing the synaptic output of the E-cells. We define 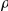 to be energy of the component of 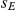 in the gamma frequency band, divided by the energy of all of 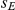; see Text S1, Section B for complete details.

## Results

### Experiments

In kainate-activated mouse hippocampal slices, expressing the light-activated cation channel ChIEF under the control of the CaMKII promoter allowed us to selectively drive CA3 pyramidal cells, without optically affecting other cell classes present in the network. We explored the effects of modulating the light intensity, focusing on two conditions: “weak” (1–3 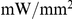, 470 nm) and “strong” (10–30 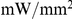, 470 nm) stimulation.

Thus there were two sources of excitation in these experiments. The first was the kainic acid in the bath, which induced a persistent gamma (25–50 Hz) oscillation in the LFP as reported by others, e.g., [14], [15]. We model the effect of the kainic acid as stochastic drive to the E-cells – see Methods and Text S1, Section B, as well as refs. [12] and [16]. The second source of excitation was optogenetic drive. We model it as tonic drive.

**Figure 1:**
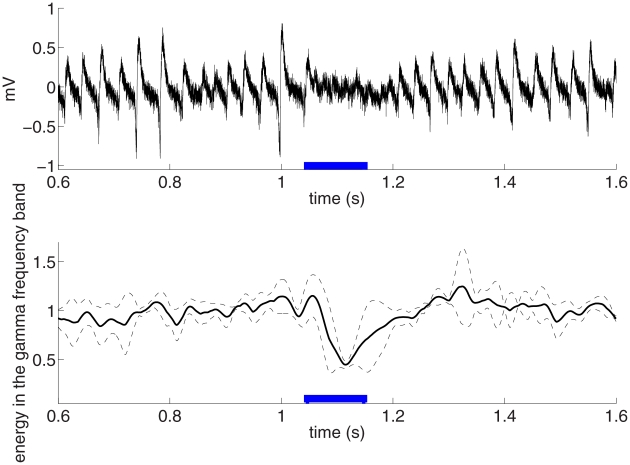
Weak light stimulation of pyramidal cells reduces gamma power. A. Raw trace of the LFP measured in the CA3 stratum radiatum of a hippocampal slice. Background drive given by 400 nM kainic acid induced gamma oscillations (peak frequency 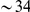 Hz. A 100 ms, weak (1–3 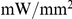) pulse of blue light, indicated in blue, reduced the amplitude of the ongoing oscillations. B. Population data from 94 trials, 8 slices plotting energy in the gamma band (25–50 Hz) as a function of time. The average energy during the stimulation period was 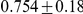 of the pre-stimulation baseline. The dashed lines indicate one estimated standard deviation.

Weak optogenetic drive reduced the power of ongoing oscillations (Fig. 1). Energy in the low gamma frequency band during weak stimulation was 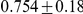 of the pre-stimulation baseline (94 trials, 8 slices, 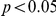), with no significant change in peak frequency (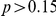). It is important to note that the stimulation did not simply result in a shift to a faster oscillation frequency, as the energy in all higher frequency bands was also decreased.

In contrast, strong optogenetic drive resulted in the emergence of fast oscillations with a peak frequency of 86.8 Hz and a standard deviation of 18.4 Hz (40 trials, 4 slices), significantly different from the baseline oscillation frequency of 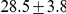 Hz (60 trials, 5 slices) (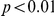). The fast oscillations appeared to replace, rather than superimpose onto, the baseline rhythm, as energy in the 25–50 Hz frequency band was 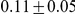 of baseline (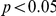).

### Modeling “weak” vs. “strong” optogenetic drive

As has been measured by others (e.g., Huber *et al.* [24, Fig. 1G]), the intrinsic variation in the amount of channelrhodopsin expressed in one cell vs. another means that stronger light will elicit spiking in more cells than weaker light. We therefore use computational simulation and mathematical analysis to study how the number of driven cells governs whether or not a PING rhythm forms in model networks.

The total number of active cells determines the strength of synaptic input per target cell. A more fundamental question is, therefore, how weakening synaptic connections leads to the disintegration of PING rhythms. We study this question first, then apply the conclusions to understanding how gamma rhythms break down when reducing the number of tonically driven E-cells, or reducing, in a spatially structured network, the size of the region in which the E-cells are driven, or making synaptic connectivity more local.

### Heterogeneity in input to I-cells matters when E-to-I-synapses are of marginal strength, not otherwise

As one gradually weakens the E-to-I-synapses in an E/I-network, there comes a point at which the PING mechanism fails. Where the breakdown occurs depends on network heterogeneity to some degree, but we show below that it can be predicted, with good accuracy, by studying homogeneous networks: In a homogeneous network, weakening of E-to-I-synapses leads to a sudden switch from 1∶1 entrainment of I-cells by E-cells to more complicated patterns, such as 2∶1 entrainment. The synaptic strength at which this happens approximately equals the synaptic strength at which rhythmicity breaks down, somewhat more gradually, in a heterogeneous network. We will also show that for significantly stronger E-to-I-synapses, even fairly substantial heterogeneities in synaptic connections and external drives to the I-cells do not have strong effects on I-cell synchrony.

#### Single-cell analysis

To support the claims in the preceding paragraph, we first consider a single I-cell, with external drive below the spiking threshold, initialized at rest. In response to an excitatory synaptic input pulse arriving at time zero, the I-cell may or may not spike; if it does, we denote the delay between the pulse arrival time and the time of the spike, measured in ms, by 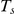. If 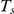 depends sensitively on the pulse strength and the external drive to the I-cell, then the response of a population of I-cells with heterogeneous external drives receiving excitatory pulses of heterogeneous strengths should be expected to be significantly spread out.

**Figure 2:**
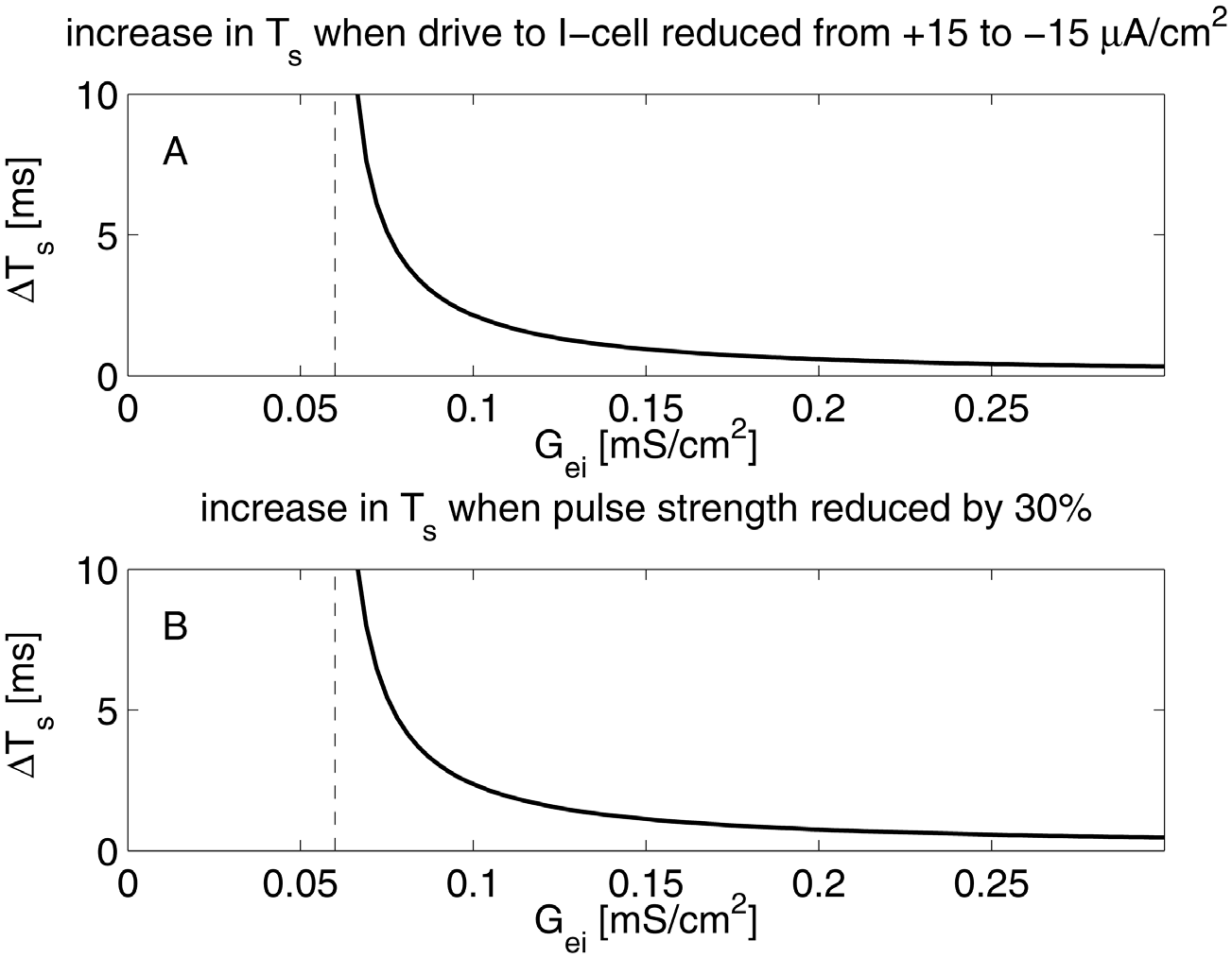
Effect of varying parameters on the delay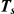 between the arrival of an excitatory synaptic input pulse to an I-cell and the resulting spike of the I-cell. A: Increase 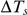 in 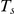 resulting from a reduction in drive density to the I-cell from 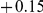 to 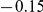 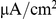. The dashed vertical line indicates the pulse strength 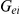 below which the pulse fails to elicit a spike when external drive density to the I-cell is 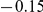 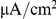. B: Increase 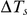 in 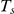 resulting from reducing pulse strength by 30%, from 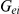 to 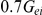. The external drive to the I-cell is fixed at zero in panel B. The dashed vertical line indicates the value of 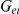 below which the pulse of strength 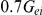 fails to elicit a spike.

Fig. 2 demonstrates that the effects of variations in the external drive to the I-cell or the input pulse strength rapidly become minor as the pulse strength rises above the strength needed to elicit a response of the I-cell. The figure shows the change 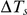 in 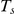 resulting from reducing the external drive to the I-cell (panel A), or reducing the strength of the pulse (panel B). In panel A, the variable 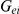 on the horizontal axis is the strength of the input pulse. In panel B, it is the strength of the stronger of the two input pulses being compared. The dashed vertical line indicates the value of 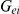 below which the I-cell fails to respond when external drive is lowered (panel A) or pulse strength is reduced (panel B). For details, see caption of Fig. 2 and Text S1, Section B. For an I-cell modeled as an integrate-and-fire neuron, a similar result is proved rigorously in Text S1, Section D.

#### Network simulations

**Figure 3:**
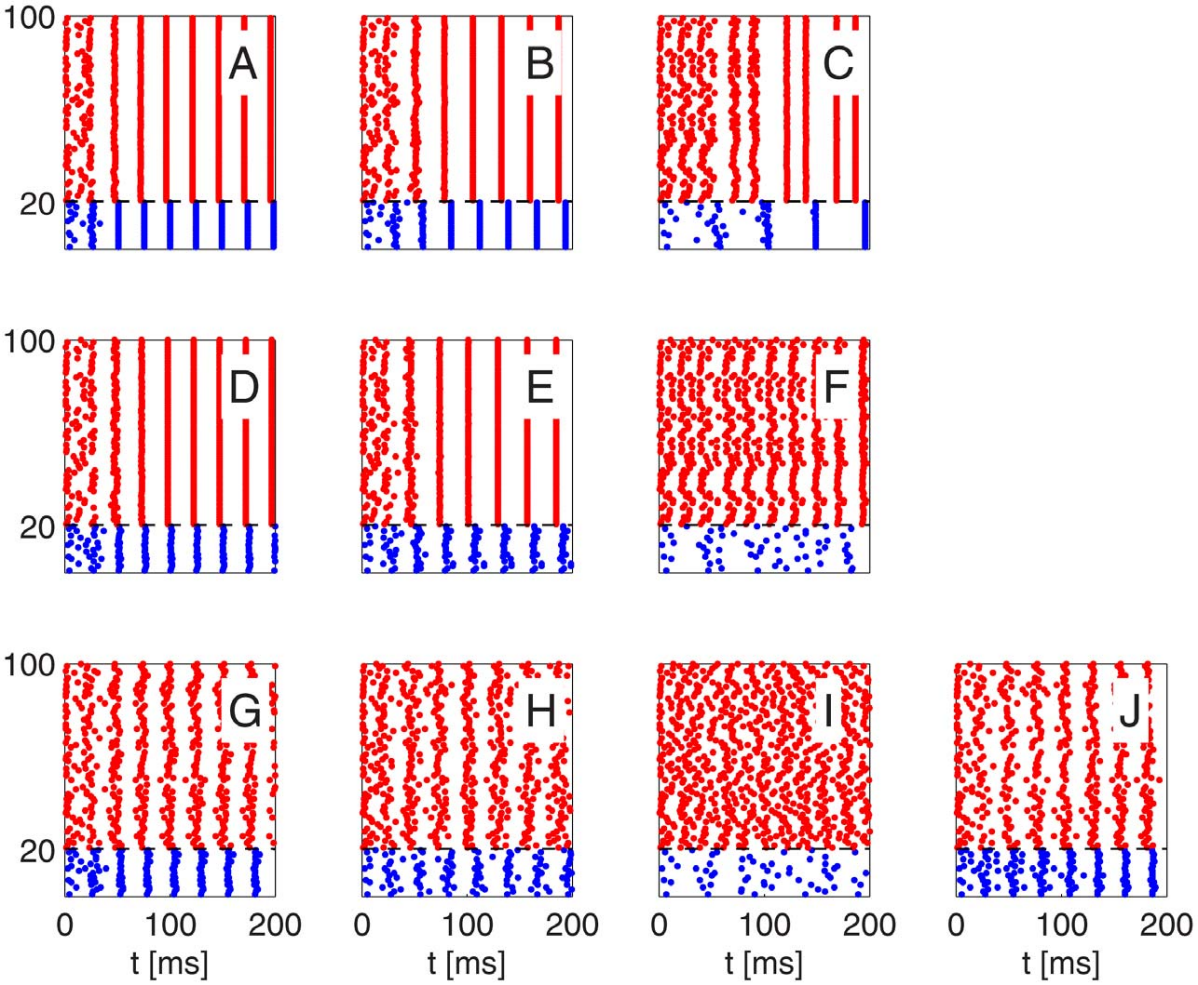
Spike rastergrams illustrating breakdown of strong PING rhythm as E-to-I-synapses are weakened. Blue dots indicate spike times of I-cells (cells 1–20), and red dots indicate spike times of E-cells (cells 21–100). From left to right: mean excitatory conductance density per I-cell 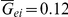 (panels A, D, G), 0.08 (B, E, H), and 0.04 (C, F, I, and J) 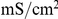. The network in panels A, B, and C is homogeneous. In panels D through F, the E-to-I-connection is sparse and random (50% connectivity). In panels G through I, the I-to-E- and I-to-I-connections are sparse and random as well (50% connectivity), and so are external drives: 15% heterogeneity in drives to E-cells, and drives to I-cells vary between 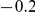 and 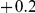 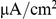. (See paragraph surrounding Eq. S8 in Text S1 for the precise meaning of “15% heterogeneity”.) The parameters in panel J are those of panel I, but mean external drive density to the I-cells is raised from 0 to 0.4 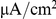, and there are no I-to-I-synapses.

In Fig. 3, we present results of network simulations leading to similar conclusions. In each case, the initialization is asynchronous. In Panels A, B, D, E, G, H, and J, a PING rhythm forms within one or two gamma periods. Panels A through C show results of simulations of networks without any heterogeneities and with all-to-all connectivity, with, from left to right, decreasing strength of E-to-I-synapses. In Panel C, the E-to-I-synapses are so weak that a single spike volley of the E-cells is no longer sufficient to trigger a spike volley of the I-cells, and 1∶1 entrainment is replaced by 2∶1 entrainment. (This is accompanied by a sudden and substantial drop in oscillation frequency.) Panels D through F show results of similar numerical experiments, with sparse, random E-to-I-synapses, but with the same values of 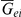. The sparseness and randomness of the E-to-I-synapses has little effect until the synaptic strength gets so low that even in the homogeneous network, 1∶1-entrainment would break down. Panels G through I show similar results again, now with sparse, random connectivities for E-to-I-, I-to-E-, and I-to-I-connections, but with the same values of 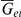, 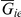, and 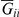 as before, and with heterogeneity in external drives. Again, the breakdown of the rhythm occurs at approximately the same point as before. It occurs not because the I-cell spike volleys spread out, but because many of the I-cells receive too little excitatory input to participate at all, weakening the inhibitory input to the E-cells to the point where its synchronizing effect is lost.

Our analysis suggests that it should be possible to restore the rhythm in Fig. 3I, for example, by raising the drive to the I-cells instead of raising excitatory conductance. This is indeed the case; see Fig. 3J. To make sure that the rhythm in Fig. 3J is not ING [11], i.e., not based on the interaction of the I-cells, we removed the I-to-I-synapses altogether in panel J of Fig. 3.

**Figure 4:**
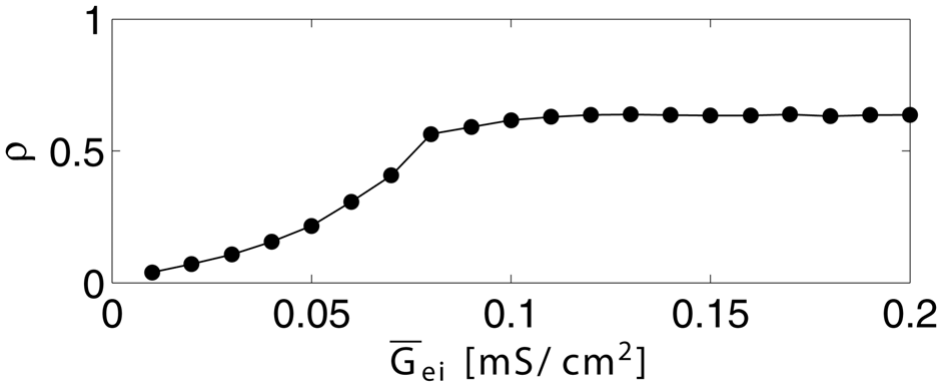
Quantitative measure of gamma rhythmicity. The measure 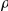 (see Eq. S10 in Text S1) is plotted as a function of mean excitatory conductance density per I-cell, 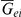, with all other parameters as in Fig. 3, bottom row of panels.

In a heterogeneous network, the loss of the gamma rhythm, as E-to-I-synapses are weakened, is gradual, not a sudden bifurcation. To demonstrate this, Fig. 4 displays our measure 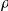 of gamma rhythmicity (see Methods and Text S1, Section B for the definition of 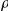) as a function of the mean excitatory conductance density per I-cell, with all other parameters fixed as in the bottom row of panels in Fig. 3.

### Heterogeneity in input to E-cells always matters, but its effects are greatest when I-to-E-synapses are of marginal strength

Weakening of the I-to-E-synapses eventually results in breakdown of the PING mechanism. As for the E-to-I-synapses, the point of breakdown can be predicted well by studying homogeneous networks. However, here the breakdown of the rhythm in the heterogeneous network is not signaled by downright breakdown in the homogeneous network, but by lengthening of the time needed to establish the rhythm in the homogeneous network when starting from asynchronous initial conditions. To demonstrate and explain this point, we will first give a computational analysis of the response of a single cell to an inhibitory pulse, then present results of network simulations. Finally, we will give a detailed analysis for a simplified problem, elucidating the reason for the link between slow convergence to synchrony in a homogeneous network and failure to synchronize at all in a heterogeneous network.

#### Single-cell analysis

We consider here the response of a single E-cell, driven above threshold, to an exponentially decaying inhibitory synaptic input pulse. A study of how the response of the E-cell depends on the timing of the inhibitory pulse yields insight into the synchronization of asynchronous populations of E-cells by inhibitory pulses.

In ref. [1], we showed that the inhibition creates an “attracting river” [25], [26] in a phase space in which one of the variables is the decaying inhibitory conductance, and that the synchronization of a population by an inhibitory pulse can be understood as a consequence of this river. Here we visualize the synchronizing effect of a single pulse of inhibition in a different way, using plots similar to those of [27, Fig. 1]. We denote by 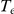 the intrinsic period of the E-cell. We assume that at time zero a spike occurs, and that an inhibitory input pulse arrives at some time 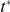 with 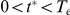. We denote by 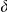 the time between the arrival of the inhibitory pulse and the next spike. If 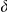 were independent of 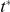, the time of the first spike following the arrival of the inhibitory pulse would be independent of the past history of the neuron; thus a single inhibitory pulse would synchronize a previously asynchronous homogeneous population of non-interacting E-cells.

**Figure 5:**
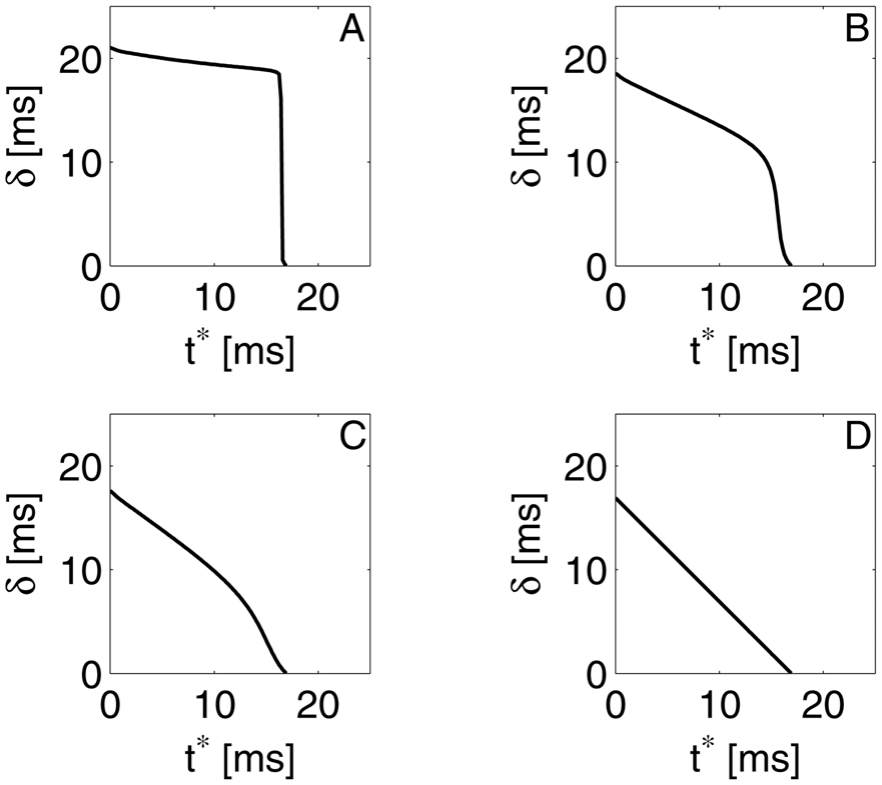
Time 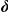 between arrival of inhibition and next spike of an E-cell, as a function of time 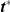 between previous spike and arrival time of inhibition. The external drive density to the E-cell is 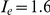 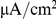, and the inhibitory conductance density 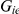 is (A) 0.24, (B) 0.12, (C) 0.06, and (D) 0 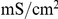.

Fig. 5 shows the computed dependence of 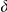 on 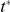. For strong inhibition (panel A), 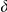 is nearly independent of 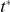. Thus a single inhibitory pulse leads to nearly perfect synchronization of a population. However, at some value of 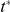 close to 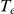, 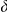 suddenly drops to near-zero; this corresponds to the fact that when the inhibitory pulse arrives very close to a spike, it cannot significantly delay the spike. In [1], we interpreted this sudden transition, for theta neurons, as the result of the crossing of an “unstable river” [25], [26]. Panels B, and C of Fig. 5 demonstrate the effect of weakening the inhibitory pulse: 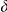 becomes significantly dependent on 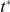 throughout the entire range, 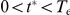. Thus a weak inhibitory pulse no longer comes close to erasing the memory of the past: A cell that was closer to spiking before the pulse arrived (larger 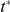) spikes earlier even in the presence of the inhibitory pulse (smaller 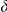). In the limiting case of an inhibitory “pulse” of zero strength, 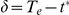, so 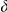 decreases linearly with 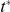 (panel D of Fig. 5).

This analysis suggests that effects of heterogeneity (in either external drive or strength of inhibition) on synchronization by an inhibitory pulse should become more pronounced as inhibition weakens: The phase dispersion caused by heterogeneity increases from cycle to cycle. We will confirm and expand upon this conclusion below.

#### Network simulations

**Figure 6:**
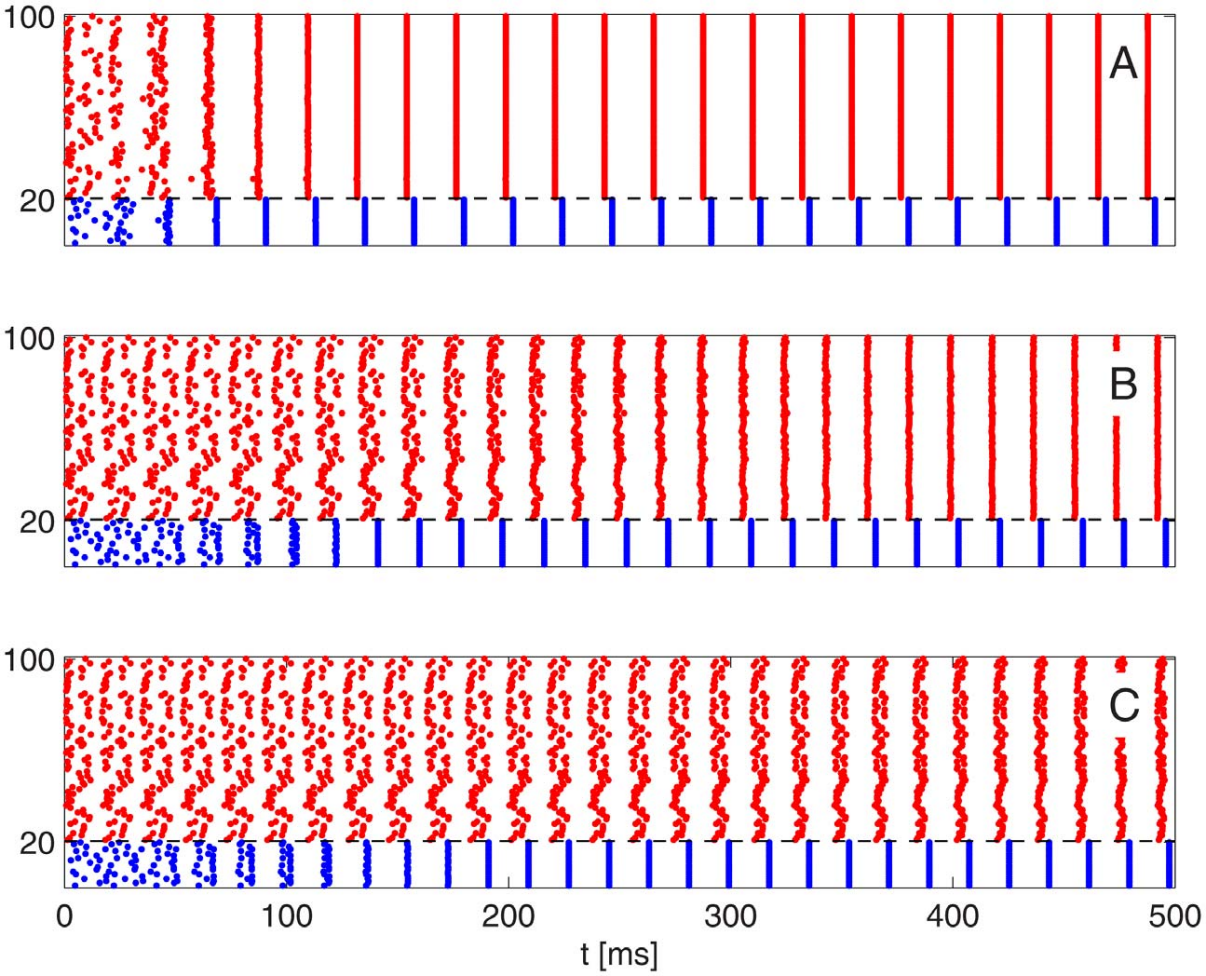
In a homogeneous network, the strong PING rhythm becomes less and less rapidly attracting as I-to-E-synapses are weakened. As before, blue dots indicate spike times of I-cells (cells 1–20), and red dots spike times of E-cells (cells 21–100). From top to bottom: 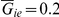, 0.05, 0.02 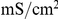.

Fig. 6 illustrates what happens as inhibition is weakened in a homogeneous network: The PING rhythm takes longer to be established. However, even for very weak inhibition, a tightly synchronous rhythm eventually emerges: Even in panel C of Fig. 6, synchronization becomes perfect, to the eye, by time 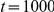 ms (not shown in the figure).

Notice that the I-cells synchronize tightly before the E-cells synchronize in Fig. 6. In general, the I-cells tend to synchronize more tightly than the E-cells in PING [1]. Their synchronization is induced by the spike volleys of the E-cells. Even when those volleys are not tightly synchronous, the I-cells all receive the same (if the E-to-I synapses are all-to-all) or nearly the same (if the E-to-I synapses are sparse, but not too sparse) excitatory input pulses, which synchronize them.

**Figure 7:**
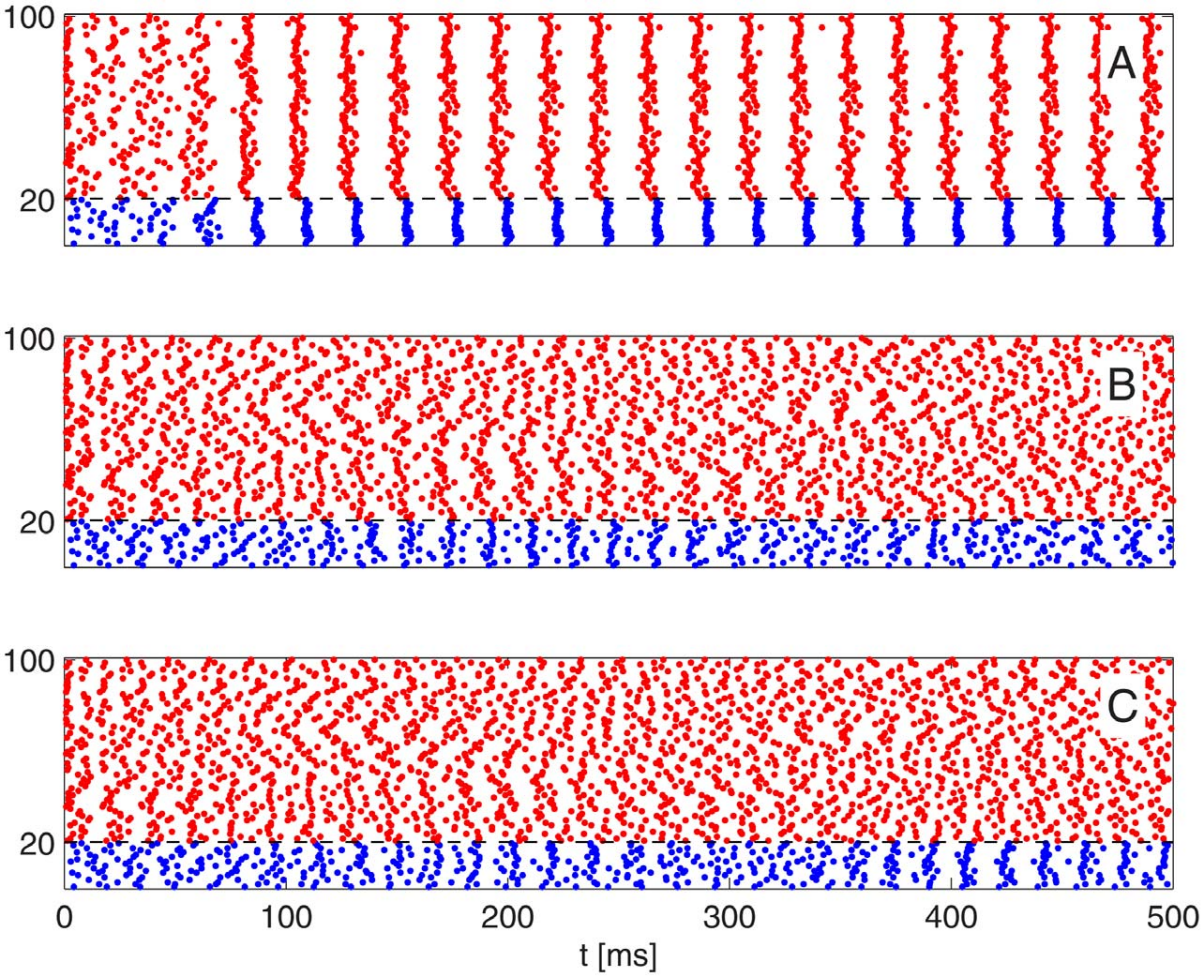
A simulation similar to that in **Fig. 6**, but with heterogeneity in drive to the E-cells, and with sparse, random E-to-I-synapses. The heterogeneity in drive to the E-cells is 10% (see paragraph surrounding Eq. S8 in Text S1). E-to-I-connections are removed with 50% probability, and those that are not removed are doubled in strength to preserve 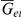 (see Text S1, Section B). Top to bottom: 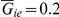, 0.05, 0.02 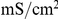. The rhythm in the I-cells seen in panel C is based entirely on the interaction of the I-cells, i.e., it is an ING rhythm [11]; see Text S1, Section F.

Heterogeneity alters the behavior in Fig. 6 dramatically. Fig. 7 shows what happens when one introduces 10% heterogeneity (see paragraph surrounding Eq. S8 in Text S1) in the external drive to the E-cells in the simulations of Fig. 6 and makes the E-to-I-synapses sparse and random (50% connection probability, with 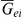 unchanged). In those cases when the PING rhythm takes some time to be established in Fig. 6, it is not established at all in Fig. 7.

The connection between loss of rhythmicity in the heterogeneous network and the inability of the homogeneous network to synchronize in a small number of cycles can be understood, in light of our earlier discussion of the synchronizing effect of a single inhibitory pulse, as follows. Inhibition is not able to overcome the effects of heterogeneity in inhibitory synaptic inputs or external drives to the E-cells [1], [28], but a strong enough pulse of inhibition removes the effects of different initial conditions on the next time to spike, allowing target cells to lock to periodic inhibitory drive with a range of phases. When inhibition is not strong enough to remove the effects of past history in one or two cycles, the spread of phases resulting from heterogeneity grows from cycle to cycle.

**Figure 8:**
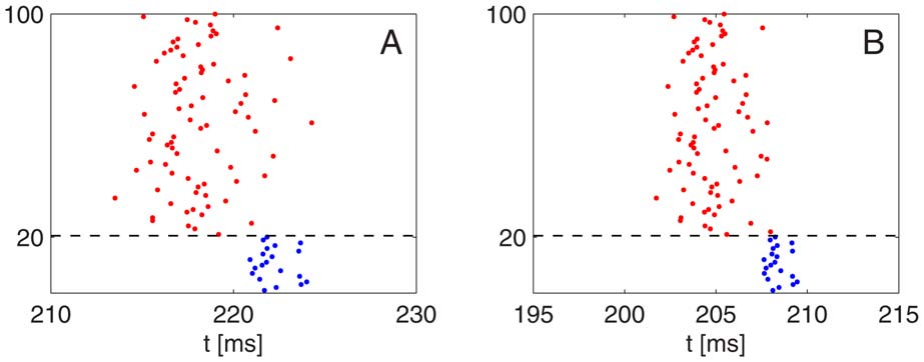
Ten-fold strengthening the I-to-E-synapses does not erase the effects of heterogeneity on E-cell synchronization. A: Close-up of Panel A of Fig. 7. B: Close-up of a simulation with 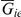 raised from 0.2 to 2.0 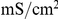, all other parameters as in Panel A of Fig. 7.

When, on the other hand, inhibition is strong enough for a PING rhythm to be established rapidly, further strengthening of inhibition does not substantially reduce the heterogeneity effect [1], [28]. This point is illustrated by Fig. 8, which shows a close-up of panel A of Fig. 7, and a close-up of a simulation with the strength of inhibition increased ten-fold, but all other parameters as in Fig. 7A. The E-cell synchronization obtained with the ten times stronger inhibition is not very much better; see also Text S1, Section E.

**Figure 9:**
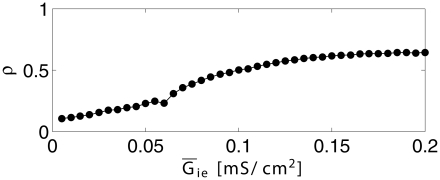
Loss of energy in the gamma range, as I-to-E-synapses are weakened, is gradual, not sudden. Measure of gamma rhythmicity, 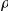 (see Eq. S11 in Text S1), as a function of 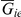, the mean inhibitory conductance density per E-cell, with all other parameters as in the bottom panel of Fig. 7.

**Figure 10:**
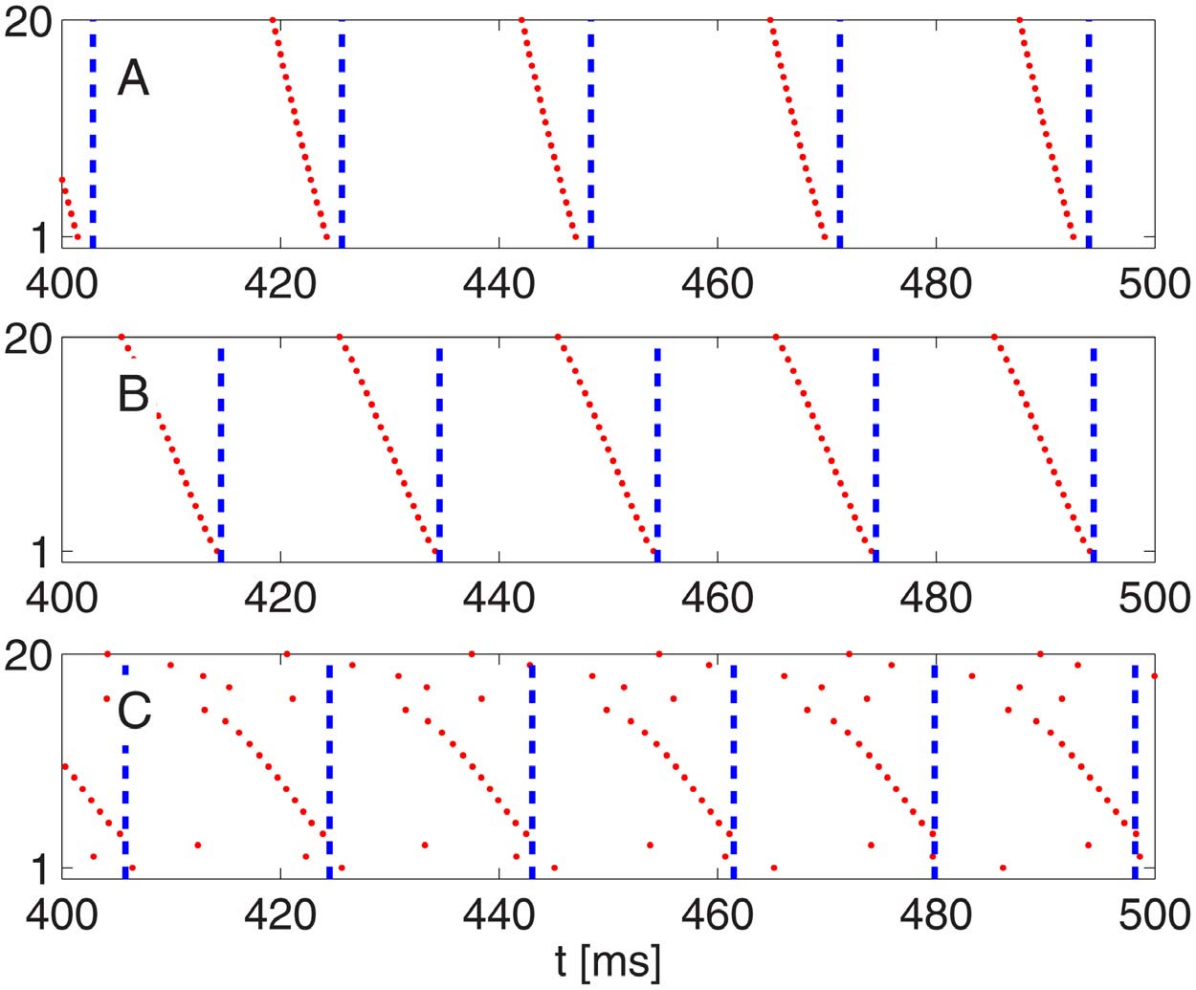
Breakdown of gamma rhythm, as inhibition is weakened, in a simplified model network. External drive to the E-cells is heterogeneous. Inhibition is strongest in panel A and weakest in panel C. The network is smaller than in earlier network simulations (20 E-cells and one I-cell), and the E-cells are put in order of linearly increasing external drive. The spike times of the I-cell are indicated by the blue dashed vertical lines. For complete details, see Text S1, Section B.

The loss of energy in the gamma frequency range, as I-to-E-connectivity is weakened in a heterogeneous network, is gradual; see Fig. 9. Note, however, the sharp kink in the graph of Fig. 9. Qualitatively, we have found this feature of the graph to be robust with respect to parameter variations, but for the complicated network underlying Fig. 9, we have not been able to explain it definitively. It seems natural to hypothesize that it reflects an analogue of the sudden transition in network behavior demonstrated, for a much simpler model network, in Fig. 10; that figure will be discussed in detail next.

#### More detailed analysis of a simpler network

The use of a simplified model network allows us to be more specific about the nature of the loss of synchrony as inhibition is weakened. We use a smaller network (20 E-cells and 1 I-cell), with drive to the E-cells linearly increasing with neuronal index, but without any other heterogeneities, and in particular with all-to-all connectivity. Fig. 10 shows how the rhythm deteriorates, and eventually breaks down, as inhibition is weakened. In Figs. 10A and B, each E-cell spikes exactly once between any two I-cell spikes, with more strongly driven E-cells spiking sooner, and the least strongly driven E-cell spiking immediately prior to the I-cell. When inhibition gets so weak that such a rhythm is no longer possible, rhythmicity breaks down altogether (Fig. 10C).

**Figure 11:**
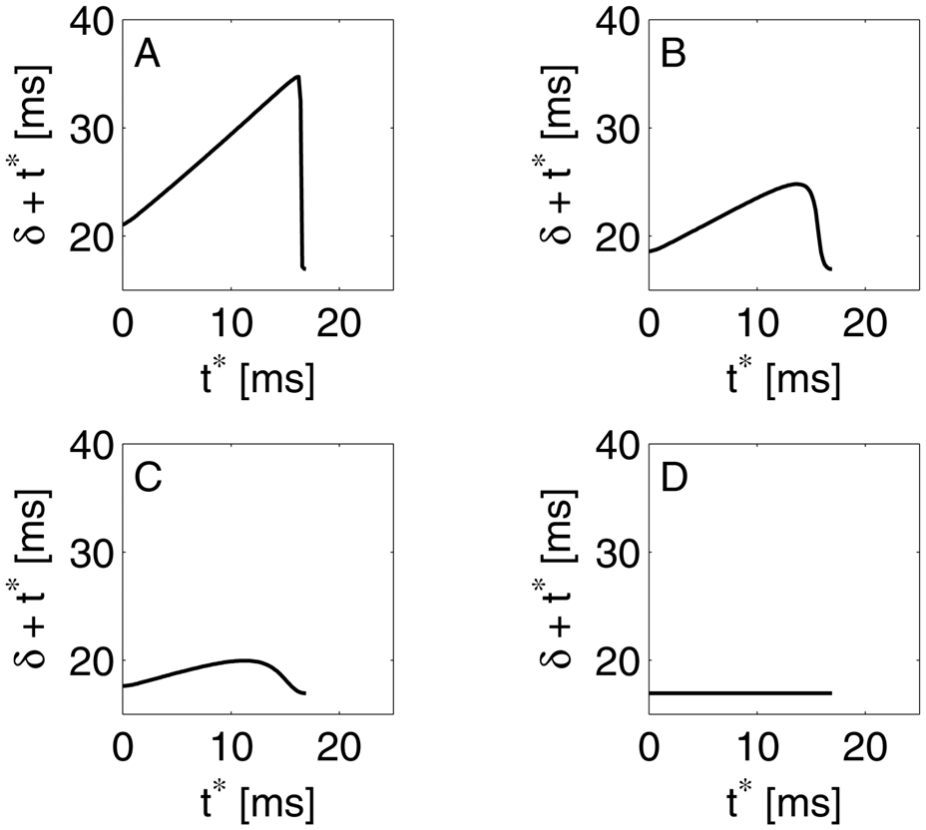
Same as **Fig. 5**, plotting 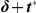 instead of 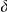.

To clarify the nature of the breakdown of the rhythm, consider again Figs. 10A and B. Denote the drive density to the 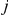-th E-cell by 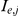. In analogy with the notation used earlier when discussing the effect of an inhibitory pulse on a single E-cell, denote the delay between the spiking of the 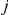-th E-cell and the next spiking of the I-cell by 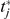. The delay 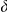 between the spiking of the I-cell and the next spiking of the 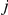-th E-cell depends on all the parameters in the network; we focus on its dependence on 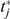 and 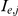 here, and therefore write 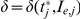. All cells in the network fire at the same period, the PING period; we denote it by 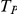. With this notation,
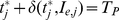
(1)for 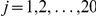. We use this equation to analyze the 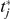. For this purpose, we plot in Fig. 11 the quantity 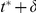 (the left-hand side of Eq. (1)) as a function of 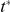. Thus Fig. 11 displays precisely the same information as Fig. 5; the only difference is that in Fig. 11, 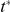 has been added to 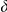. Recall that in Fig. 5, tight synchronization of a homogeneous population by a single pulse is reflected by a nearly horizontal graph over most of the range of values of 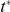. In Fig. 11, this corresponds to a slope close to 1 over most of the range of values of 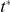.

**Figure 12:**
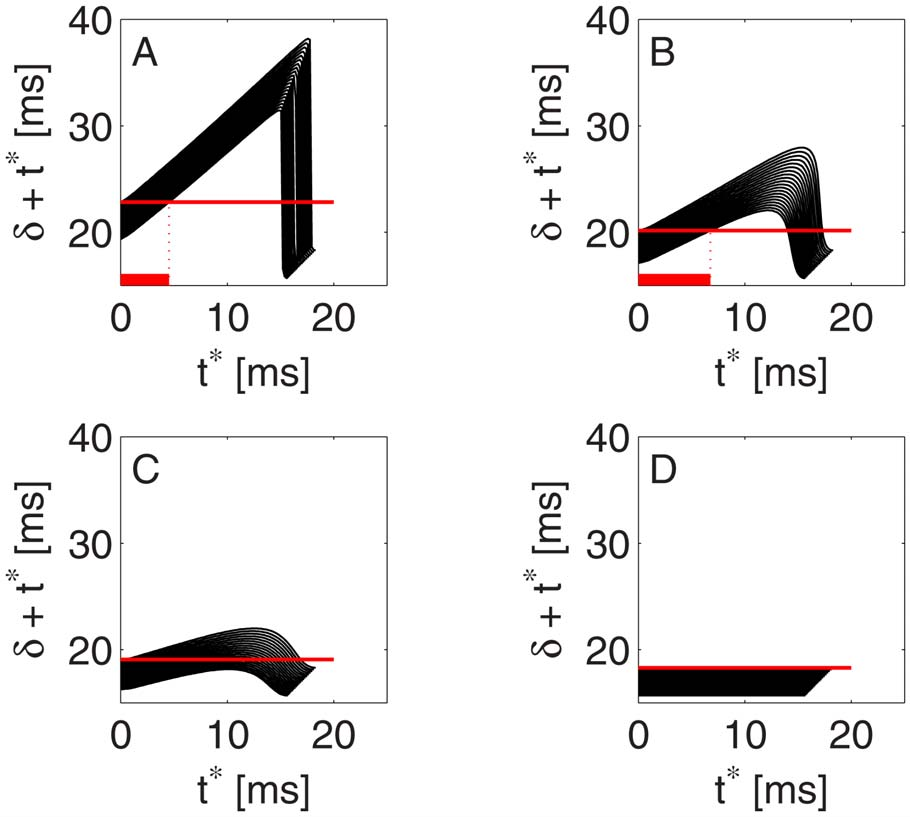
Same as **Fig. 11**, plotting the graphs for a range of values of external drives. The drive 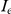 to the E-cells varies between 1.4 and 1.8. The bold horizontal red lines in panels A and B indicate the duration of the E-cell spike volley. In panel C, the inhibition is so weak that a rhythm in which each E-cell participates exactly once per cycle no longer exists. Panel D shows the limiting case of no inhibition.

For each 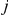, 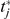 satisfies Eq. (1). In Fig. 12, we therefore plot 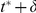 for all 20 values of 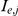 used in our model network simultaneously. According to Eq. (1), the values of 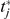 are obtained by intersecting, in each of the panels of Fig. 12, the 20 graphs shown with a single horizontal line. This horizontal line is indicated in red in Fig. 12, assuming that (as in panels A and B of Fig. 10) the I-cell spikes immediately after the first (least strongly driven) E-cell, i.e., 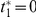. Panels A and B of Fig. 12 show that for each 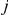, Eq. (1) has two solutions 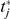; we select the smaller of the two solutions because in a PING rhythm, the inhibitory spike volleys rapidly follow the excitatory ones. Panels A and B of Fig. 12 also show the duration of the E-cell spike volleys (bold red lines). In panel C, the maximum of 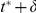 for the most strongly driven E-cells (lower edge of the band shown in panel C) is smaller than the minimum of 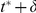 for the least strongly driven E-cells (upper edge of the band). This implies that a rhythm in which each E-cell participates exactly once per period, i.e., a phase-locked solution, does not exist in this case; the slope of the ascending branch of the curves is not steep enough because inhibition is too weak.

Thus the condition for rapid synchronization of the E-cells in a homogeneous network, namely that the slope of the ascending branches of the curves in Figs. 11, 12 be close to 1, turns out to be precisely the condition under which rhythms of the kind shown in Figs. 10A and B can exist, explaining why heterogeneity destroys the rhythm altogether when, in a homogeneous network, it takes many gamma cycles to synchronize the E-cells.

### Specific drive to too small an ensemble can abolish even the background weak PING rhythm

We now connect the previous results to the size of cell assemblies. We start with a spatially unstructured network that is sparsely and randomly connected, and consider the behavior as the number of tonically driven E-cells is reduced. Our expectation for what should happen comes from our earlier discussion of weakening E-to-I-coupling; reducing the number of active E-cells reduces the total excitatory current received by I-cells. Thus we expect the rhythm to be destroyed if the number of participating E-cells gets too small.

**Figure 13:**
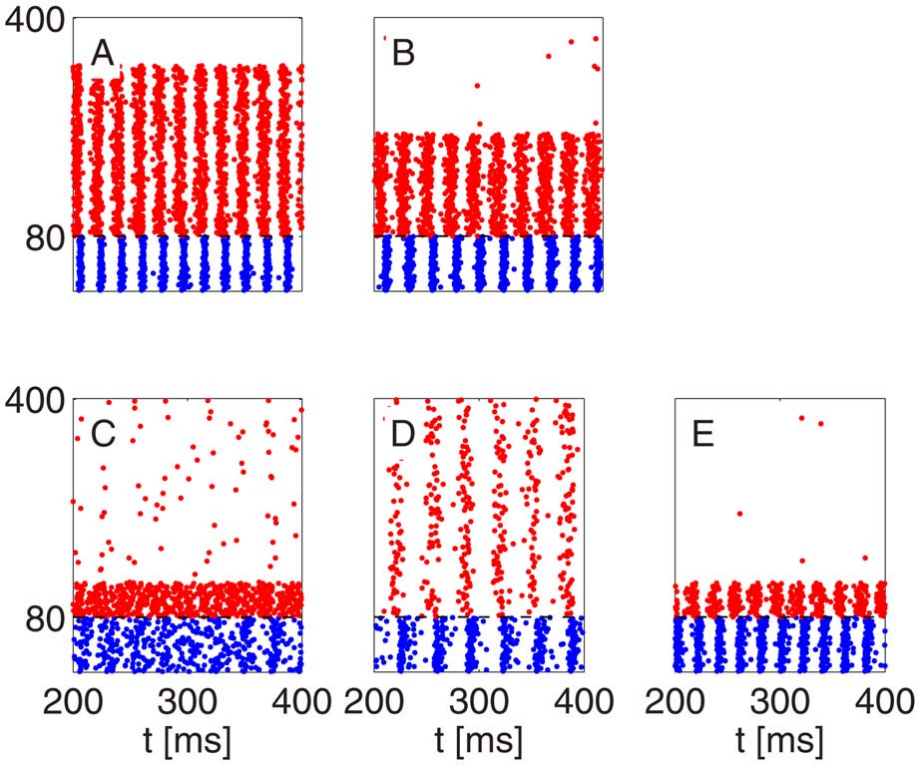
Breakdown of strong PING as the number of E-cells receiving strong, time-independent drive is reduced. Neurons 1–80 are I-cells, and neurons 81–400 E-cells. All E-cells receive stochastic drive. In addition, 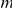 E-cells receive strong tonic drive, with 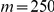 (A), 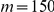 (B), 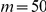 (C), and 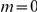 (D). Rhythmicity is largely abolished when 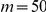 (panel C), but weak PING emerges when 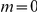 (panel D). In Fig. 13C, the rhythm returns when the strength of the E-to-I-synapses is tripled (panel E).

Fig. 13 shows the breakdown of the rhythm. In contrast with other simulations in this paper, in the simulation of Fig. 13, all E-cells receive a background of stochastic input; see Text S1, Section B for details. As the number of E-cells receiving tonic drive is reduced, there is a transition from PING (A and B), through an arrhythmic regime in which both E- and I-cells continue spiking (C), to a background weak PING rhythm [12] driven by the stochastic input to the E-cells, akin to a kainate-induced persistent gamma rhythm in a hippocampal slice. We hypothesize that panel D of Fig. 13 is an analogue of what happens in Fig. 1 before and after the stimulus. As discussed earlier, we think of driving fewer E-cells as the analogue of using weaker optogenetic stimulation; therefore panel C is an analogue of what happens in Fig. 1 during the stimulus. We show the time interval from 200 ms to 400 ms, not the initial time interval from 0 ms to 200 ms, in Fig. 13 in order to demonstrate that in panel C, the rhythm is not just slow to arise, but does not arise at all.

The mean frequency of the I-cells is approximately 54 Hz in panel A, 48 Hz in panel B, and 29 Hz in panel C. Panel D of Fig. 13 shows a simulation in which no E-cells receive tonic drive; the results would look very similar if, for instance, 5 E-cells received tonic drive. When a significant number of the E-cells receive specific drive (panels A–C), the weak PING rhythm in the other E-cells is suppressed either by fast rhythmic activity of the I-cells (panels A and B), or by sparser, but arrhythmic and therefore still powerful [2] activity of the I-cells (panel C).

With fewer E-cells driven tonically, the I-cells receive less synaptic excitatory input; therein lies the main connection between the simulation results in Fig. 13 and the earlier ones concerning the effect of weakening E-to-I-synapses (Fig. 3). However, effective heterogeneity in excitatory synaptic input per I-cell also becomes greater when the input originates from a smaller number of E-cells, for statistical reasons: When the excitatory synaptic input into the I-cells originates primarily from a small number of tonically driven E-cells, the heterogeneity resulting from the randomness of the connectivity is substantial. This effect is reduced by the Law of Large Numbers as the number of tonically driven E-cells increases.

In Fig. 13C, the I-cells inhibit each other. However, the I-to-I-interactions alone are not sufficient to create synchrony, i.e., there is no ING [11] rhythm, because of the heterogeneity of the drive from the E-cells [29].

Our reasoning suggests that in Fig. 13C, the rhythm should be restored if the E-to-I-synapses are strengthened: The loss of rhythmicity occurs simply because E-to-I-connectivity is so weak that more than 50 E-cells are required to sustain the rhythm. Indeed, if the strength of excitatory synapses is tripled in Fig. 13C, the rhythm does return; see Fig. 13E.

### Specific drive to too small a patch in a spatially structured network fails to elicit a PING rhythm

**Figure 14:**
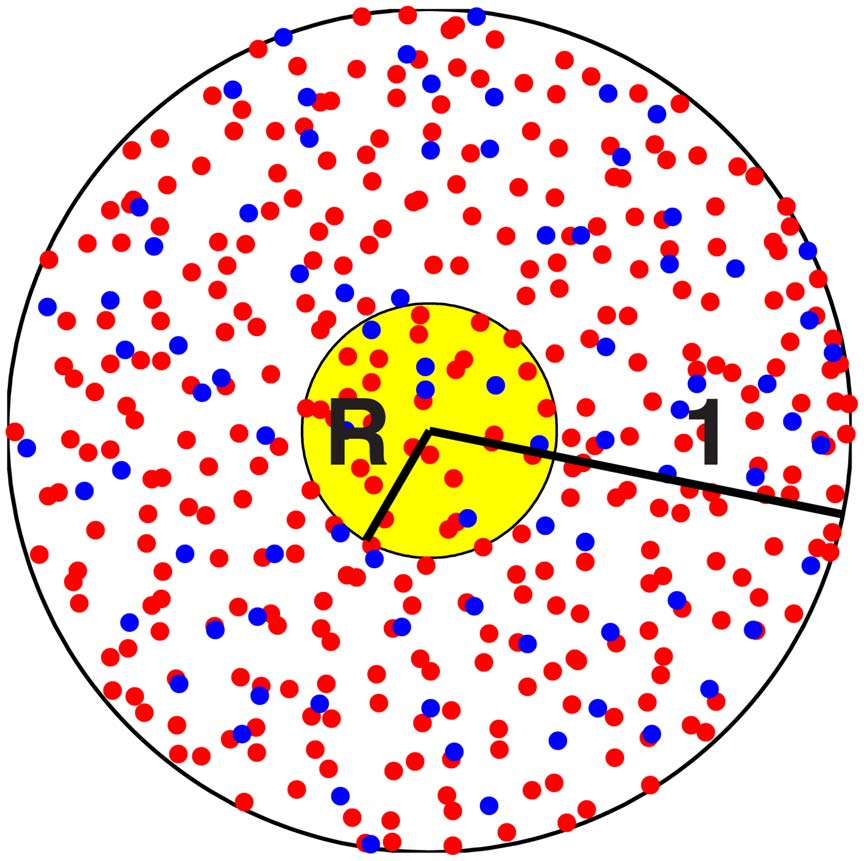
E-cells (red) and I-cells (blue) placed at random in the unit disk. E-cells in the center disk (yellow) of radius 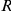 are given additional drive.

**Figure 15:**
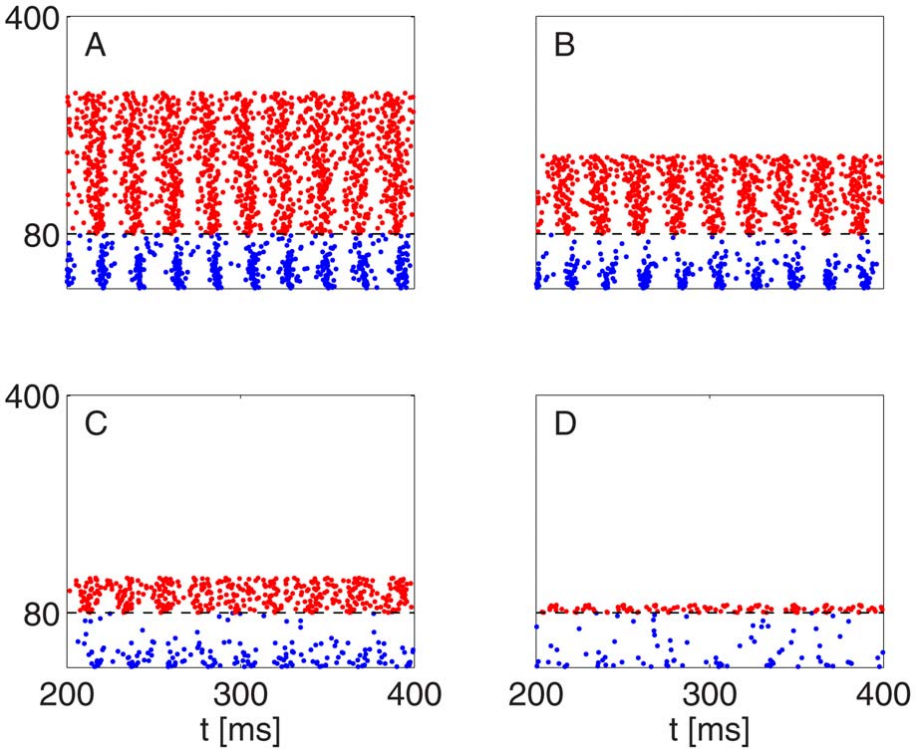
Breakdown of gamma rhythms as the radius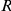 of the driven patch of E-cells is reduced. 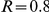
 (A), 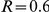 (B), 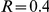 (C), and 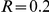 (D). The length scale 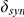 characterizing the decay of connection probability with spatial distance (see Text S1, Section, Eq. S12) equals 0.25 here. (Distance is non-dimensionalized.)

We now consider a network in which the connection probability decays with increasing distance between neurons (see Text S1, Section B). Fig. 14 shows the placement of neurons in the unit disk. (Distance is non-dimensionalized here.) Strong drive is given to the E-cells in a smaller circle of radius 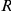, also indicated in Fig. 14. As 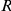 is reduced, the rhythm eventually breaks down, as shown in Fig. 15. In plotting the spike rastergrams, each of the two cell populations was ordered in such a way that smaller indices correspond to positions closer to the center of the disk.

As the size of the driven patch gets smaller, the I-cells receive less excitatory input. This is one way in which the results of Fig. 15 are related to the earlier ones on weakening E-to-I-synapses (Fig. 3) and reducing the number of cells driven (Fig. 13). In addition, however, as the driven patch decreases in size, fewer I-cells receive enough excitatory synaptic input to participate in the rhythm, since the probability of synaptic interaction decays with distance here. Thus, in fact, the loss of rhythmicity in Fig. 15 is also related to a reduction in the total amount of inhibitory input per E-cell; compare Fig. 7.

As in the earlier Figs. 3, 7, and 13, the loss of rhythmicity in Fig. 15 results from a combination of weak effective synaptic interactions and heterogeneity. There are two sources of effective heterogeneity here. The first is statistical: When fewer E-cells give synaptic input to the I-cells (or vice versa), there are more statistical fluctuations, because the Law of Large Numbers does not wash them out. If we increased the size of the model network, while proportionally reducing the strength of each individual synapse and leaving all other parameters fixed, the statistical heterogeneity would be reduced. However, here there is a second, geometric source of heterogeneity: Cells that lie near the edge of the driven patch receive fewer synaptic inputs than ones that are far from the edge. For a smaller patch, the percentage of cells significantly affected by this effect is greater. If we increased the size of the model network, while proportionally reducing the strength of each individual synapse and leaving all other parameters fixed, the geometric heterogeneity would not disappear.

**Figure 16:**
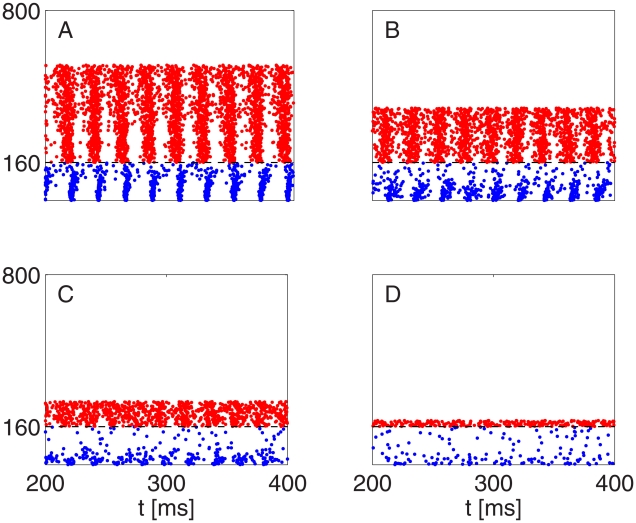
As **Fig. 15**, with numbers of E- and I-cells doubled, and strengths of individual synapses halved. The breakdown of the rhythm occurs near the same value of 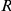.

**Figure 17:**
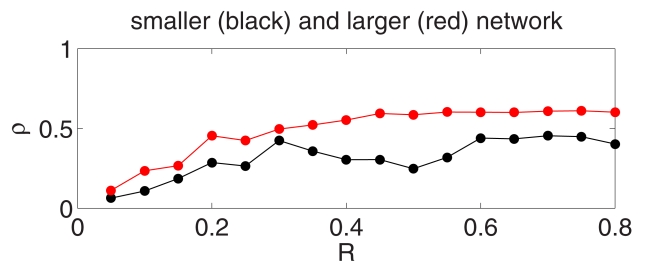
Synchrony measure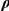 as a function of radius 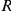 of driven patch of E-cells in **Figs. 15** and **16**. See Text S1, Section B for definition of 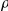.

Fig. 16 shows the same numerical experiment as Fig. 15, but with twice as many E- and I-cells, and with the strengths of individual synapses halved. Inspection of the rastergrams suggests that synchrony is sharper in Fig. 16 than in Fig. 15. This is confirmed by Fig. 17, which shows the synchrony measure 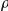 (see Text S1, Section B) as a function of the radius 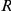 of the driven patch of E-cells. The enhanced synchrony for the larger network is likely due to the reduction in statistical fluctuations in the stochastic input per cell as the network is enlarged. Fig. 17 also shows that for both networks, there is a gradual but marked deterioration of gamma power as 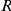 is reduced, setting in at approximately the same value of 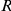.

### Specific drive to a patch in a spatially structured network fails to elicit a PING rhythm when synapses are too local

**Figure 18:**
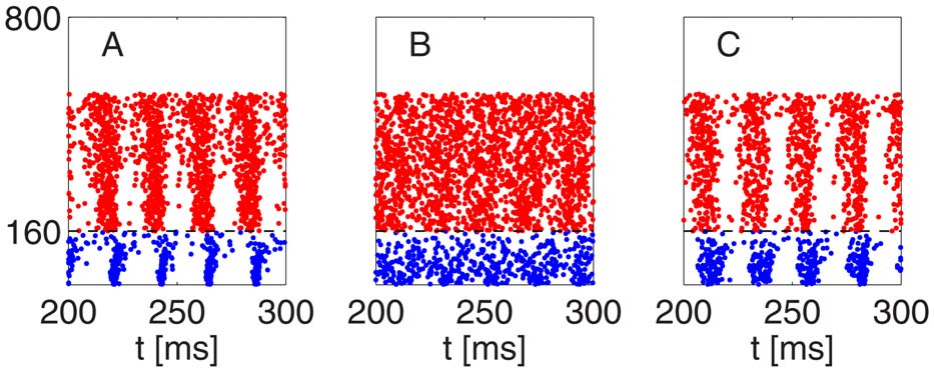
Breakdown of gamma rhythms as the synapses become too local. 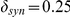
 (panel A), and 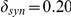 (panel B), where 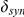 denotes the length constant characterizing the decay of the connection probability with distance (see Text S1, Eq. S12). In panel (C), 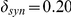 as well, but the synaptic strengths have been tripled, and the rhythm is restored.

We consider the same kind of spatially structured networks as before. However, we now fix the radius 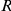 of the circular patch in which the E-cells receive specific drive, and vary the length constant 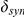 characterizing the decay of the connection probability with distance (see Text S1, Eq. S12). The effect of a decrease in 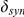 is a reduction in total synaptic input per cell, and therefore eventually the breakdown of the PING rhythm, for the reasons discussed earlier; see Fig. 18, panels A and B. Panel C of Fig. 18 shows the result of making connectivity more local, as in panel B of the figure, but compensating by strengthening the synapses: The rhythm returns. Thus panel C illustrates that the rhythm breaks down in panel B because there is too little overall synaptic input per cell, not because connectivity is too local *per se*.

## Discussion

We have examined how the PING mechanism breaks down as excitatory and/or inhibitory synapses are weakened, as the numbers of participating excitatory and/or inhibitory cells get too low, or as synaptic connectivity becomes too local. Although the breakdown of the rhythm results from the interaction of weakness of synaptic inputs with network heterogeneity, the point at which it occurs can be predicted, with good accuracy, by studying homogeneous networks. The effects of heterogeneity in synaptic or external drive to the I-cells disappear rapidly as the strength of excitatory synapses increases, and is substantial only if the excitatory synapses are marginal, i.e., nearly so weak that in a homogeneous network, 1∶1-entrainment of E- and I-cells would be replaced by a more complicated pattern such as 2∶1-entrainment. In contrast, the effects of heterogeneity in synaptic or external inputs to the E-cells remain sizable even in the limit as the strength of the inhibitory synaptic input per E-cell tends to infinity. They increase greatly, and quickly lead to a complete breakdown of the rhythm in a heterogeneous network, when inhibitory synaptic inputs into the E-cells become so weak that, in a homogeneous network, the rhythm would only be established after multiple gamma cycles. In a realistically heterogeneous network, a PING rhythm is either established rapidly, within a small number of gamma cycles, or not at all.

As the number of driven cells is reduced, the synaptic input per cell is reduced as well. At the same time, the effective heterogeneity in synaptic connections becomes more significant. The combination of effectively weaker synaptic interactions with greater heterogeneity eventually leads to the breakdown of the rhythm.

There are two reasons why a reduction in the number of driven cells can lead, in effect, to more significant heterogeneity. First, when connectivity is sparse and random, different cells receive different numbers of synaptic inputs. When many cells participate in the rhythm, this effect is largely erased by the Law of Large numbers. However, when only a small number of cells participate, it can be substantial. Second, when only the neurons in a certain spatial domain are driven strongly, and when the probability of synaptic connections decreases with increasing spatial separation, those neurons near the edge of the spatial domain receive less synaptic input than those near the center. As the size of the driven domain is reduced, the fraction of cells that are close enough to the edge for this effect to matter increases.

Our results imply that for given synaptic strengths, cell assemblies cannot be arbitrarily small. This is complementary to recent work by Oswald *et al.* [30], who have pointed out a reason why there may be an upper bound on the possible size of cell assemblies. The lower bound on the size of cell assemblies substantially depends on the strength of E-to-I- and I-to-E-coupling; stronger synapses allow smaller assemblies. To some extent, it also depends on network heterogeneity, with less heterogeneity allowing smaller assemblies.

It would be very interesting to give a specific, numerical answer to the question “How many cells must an assembly include to oscillate at gamma frequency?” Unfortunately, such an estimate would have to remain highly speculative at this point. We would, for instance, have to know how many synchronous excitatory synaptic inputs a fast-spiking parvalbumin-positive interneuron must receive for a spike to be elicited, what are the density and spatial reach of synaptic connections from pyramidal cells to fast-spiking interneurons and vice versa, and how many inhibitory synaptic inputs are required to create the synchronizing “river” in the pyramidal cells. None of these data are known with any degree of certainty, and they are surely different in different parts of the brain. These uncertainties render any attempt to give a numerical estimate futile.

In those of our simulations that involved space-dependent connectivity probabilities, we assumed that excitation and inhibition reached equally far. While this assumption is in agreement with what Adesnik and Scanziani [31] have found in their recent work for horizontal connectivity in layer 2/3 of mouse somatosensory cortex, it is not crucial for the results of the present paper.

In this study, we have focused on strong PING, i.e., PING driven by tonic excitation of the E-cells. (The only exception is Fig. 13, where we added stochastic drive to the E-cells in order to demonstrate that the weak PING rhythm created by such drive is abolished when a small, but not extremely small number of E-cells receive tonic drive.) However, we do not see a reason why similar results should not hold for stochastically driven PING rhythms.

Spencer [32] has previously studied the effects of weakening synaptic connections in E/I networks of integrate-and-fire neurons, and found rapid deterioration of gamma rhythms with the weakening of fast synaptic connections. The loss of gamma rhythmicity in [32] appears to be faster than in our numerical experiments; compare for instance [32, Fig. 3] with Fig. 4 of this paper. This is a quantitative (not qualitative) difference that may be accounted for by several differences in details between the model of [32] and ours. For instance, external drive in [32] fluctuates stochastically in time, whereas in our model the primary drive is tonic, but often with significant heterogeneity.

We note that Fig. 1 of [32] does appear to show a gradual slide into PING, contrary to our conclusion that in a heterogeneous network, PING is formed either rapidly, or not at all. However, note that the rhythm in [32, Fig. 1] relies on recurrent, NMDA-receptor-mediated excitation among the E-cells. This excitation has to build up before the rhythm begins. Here we have only considered rhythms sustained by external drive to the E-cells.

There is a considerable body of work on the stability of asynchronous states in neuronal networks [33]–[36]. Our focus here has not been on this bifurcation, in which oscillations are created, but rather on the gradual tightening of synchronization, and resulting rise in oscillation amplitude, as synaptic strengths are increased beyond the level necessary for the asynchronous state to loose its stability. (Of course, in a heterogeneous E/I network, synchrony will never get entirely tight – there is no strictly synchronous state in such a network.)

There is also previous literature on the dependence of synchronization on connectivity, e.g., [37], [38]. Our focus here is different, however; we point out that making synaptic connectivity more local, without compensating by strengthening individual synapses, can lead to a loss of rhythmicity simply because synaptic interactions get too weak, before they get too local (independently of their strength); see Fig. 18.

Bartos *et al.* [39] have suggested that the inhibitory synapses relevant to gamma oscillations should be faster and less hyperpolarizing than the ones used here. There is controversy over which are the biologically realistic parameter choices. For example, Cobb *et al.* [40] reported 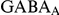-receptor-mediated inhibition in hippocampus to be hyperpolarizing, not shunting. Traub *et al.* [6] reported IPSCs with decay time constants around 10 ms during ongoing gamma oscillations. Gamma rhythms are possible in our model networks with the parameters used by Bartos *et al.*, but many of their properties, including the mechanisms by which they are lost when synaptic interactions are weakened, are different.

The fact that the PING rhythm eventually breaks down as synaptic interactions become weaker is, of course, obvious, but it suggests explanations for several seemingly unrelated recent experiments. First, in our own experiments in kainate-bathed slices of area CA3 of mouse hippocampus, strong light activation of pyramidal cells elicits a strong, fast rhythm. Weak light activation not only fails to elicit such a rhythm, but also abolishes the kainate-induced persistent gamma rhythm. We hypothesize that weaker light activation in these experiments results in activation of a smaller number of cells. These results are then closely analogous to our Fig. 13, where we examined the loss of the gamma rhythm as the number of E-cells with specific drive becomes too small. Second, in macaque primary visual cortex, gamma rhythms are elicited by spatially extended stimuli, but not by ones that are too small [18, Fig. 1]; this is expected from our Fig. 15. We note that gamma rhythms have been associated with binding [41]–[43], and may therefore not be needed for highly spatially focused neuronal activity. Third, attention has been found to reduce gamma power in primary visual cortex [19]. Our Fig. 18, which shows the loss of gamma rhythms as synaptic connections become too local, offers a possible theoretical explanation of this result, since the cholinergic modulation associated with attentional processing is thought to reduce the efficacy of lateral synaptic connections [44]. Our simulation results predict that in all of these experiments, when the rhythm disappears, vigorous activity should continue in the driven E-cells and in nearby I-cells.

In summary, the strengths of the reciprocal synaptic interactions between principal cells and those inhibitory interneurons participating in the gamma rhythm play a crucial role in determining the possible sizes of cell ensembles oscillating at gamma frequency, with stronger synapses allowing smaller ensembles.

## Supporting Information

Text S1 **This file contains all supporting information as a single PDF document.**
(PDF)
