# Supplementary material for: Minimal Size of Cell Assemblies Coordinated by Gamma Oscillations
Source: PLoS Comput Biol. 2012 Feb 9;8(2):e1002362. doi: 10.1371/journal.pcbi.1002362 (PMC3276541; doi:10.1371/journal.pcbi.1002362)
Supplement: Text S1 — This file contains all supporting information as a single PDF document. (PDF) [file pcbi.1002362.s001.pdf]

Supporting Information

A. Details of experimental methods. All chemicals, unless otherwise specified, were from Sigma-Aldrich. Lentivirus carrying the ChIEF-GFP construct [4] under control of the CaMKII promoter was stereotactically injected into the CA3 region of the hippocampus 3–6 weeks prior to the experiments. Hippocampal slices, 450 μm thick, cut in the horizontal plane, were then obtained from C57BL/6 mice that had undergone viral injection. The solution used during recording contained 126 mM NaCl, 3 mM KCl, 1.25 mM NaH2PO4, 2 mM CaCl2, 2 mM MgSO4, 24 mM NaHCO3, and 10 mM glucose, saturated with 95% O2 and 5% CO2. Experiments were performed in an interface chamber kept at 34°C. After letting the slices recover in the interface chamber for 15 minutes, kainic acid (Cayman Chemical) was bath applied at a concentration of 400 nM. Light pulse trains were synthesized by custom software written in Matlab (Mathworks), then played back via a DG-4 optical switch with a 300 W xenon lamp (Sutter Instruments) and GFP filter set (Chroma). Pulled glass electrodes placed in the CA3 stratum radiatum were used for all local field potential (LFP) recordings.

The spectral analysis of the LFP recordings was conducted off-line. To calculate the evolution of spectral energy in the 25–50 Hz range over time, the signal was band-passed in the 25–50 Hz range, using the "eegfilt" routine in the EEGLAB package (Schwartz Center for Computational Neuroscience), and the instantaneous energy estimated by taking the L^2-norm of the analytic signal. The resulting energy vs. time profile was calculated for each trial and then pooled for subsequent analysis.

The peak frequencies of the baseline and of the optogenetically induced oscillations were determined from the power spectral density of (detrended, but not filtered) LFP data. Power spectral densities were estimated using the "pwelch" routine in Matlab, which implements Welch's method [8].

B. Modeling details. Each figure of this paper is generated by a stand-alone Matlab program, available from the first author upon request. Here we give the full details of the models underlying the figures in the main text.

Figure 2: The I-cell is a Wang-Buzsáki model neuron [7]:

$$C\frac{dv}{dt} = g_{Na}m_\infty(v)^3h(v_{Na}-v)+g_Kn^4(v_K-v)+g_L(v_L-v)+I_i, \quad \text{(S1)}$$

$$\frac{dh}{dt} = \frac{h_\infty(v)-h}{\tau_h(v)}, \quad \text{(S2)}$$

$$\frac{dn}{dt} = \frac{n_\infty(v)-n}{\tau_n(v)}, \quad \text{(S3)}$$

with

$$
\begin{aligned}
x_\infty(v) &= \frac{\alpha_x(v)}{\alpha_x(v)+\beta_x(v)} \quad \text{for } x=m,h, \text{ or } n, && \text{(S4)}\\
\tau_x(v) &= \frac{0.2}{\alpha_x(v)+\beta_x(v)} \quad \text{for } x=h \text{ or } n, && \text{(S5)}\\
\alpha_m(v) &= \frac{0.1(v+35)}{1-\exp(-(v+35)/10))},\\
\beta_m(v) &= 4\exp(-(v+60)/18),\\
\alpha_h(v) &= 0.07\exp(-(v+58)/20),\\
\beta_h(v) &= \frac{1}{\exp(-0.1(v+28))+1},\\
\alpha_n(v) &= \frac{0.01(v+34)}{1-\exp(-0.1(v+34))},\\
\beta_n(v) &= 0.125\exp(-(v+44)/80)\ .
\end{aligned}
$$

Here v denotes voltage in mV, t denotes time in ms, and I denotes current density in μA/cm^2. The parameters are $C = 1$ μF/cm^2, $g_{Na} = 35$ mS/cm^2, $g_K = 9$ mS/cm^2, $g_L = 0.1$ mS/cm^2, $v_{Na} = 55$ mV, $v_K = -90$ mV, and $v_L = -65$ mV.

The excitatory pulse is assumed to arrive at time 0, modeled by adding the term

$$-G_{ei}e^{-t/\tau_e}v$$

to the right-hand side of Eq. S1, with $\tau_e = 3$ ms and $G_{ei} > 0$. (The added term drives v towards 0 mV, hence it is excitatory.) We denote by T_s the time at which the I-cell spikes; throughout the paper, we take the "spike time" to be the time when the membrane potential v rises above 0 mV. Since T_s is a function of G_{ei} and the external drive I_i, we also write $T_s = T_s(G_{ei}, I_i)$. With this notation, panel A of Fig. 2 shows $T_s(G_{ei}, -0.15) - T_s(G_{ei}, 0.15)$, as a function of G_{ei}, and panel B shows $T_s(0.7G_{ei}, 0) - T_s(G_{ei}, 0)$, as a function of G_{ei}.

Figure 3: The I-cells are as in Fig. 2. The E-cells are as in ref. [5]. Eqs. (S1)–(S4) are as in the I-cell model, with I_i replaced by I_e in Eq. (S1). Eq. (S5) is replaced by

$$\tau_x(v) = \frac{1}{\alpha_x(v)+\beta_x(v)} \quad \text{for } x=h \text{ or } n. \qquad \text{(S5)}'$$

The rate functions α_x and β_x, $x = m, h$, and n, are

$$
\begin{aligned}
\alpha_m(v) &= \frac{0.32(v+54)}{1-\exp(-(v+54)/4)}, \\
\beta_m(v) &= \frac{0.28(v+27)}{\exp((v+27)/5)-1)}, \\
\alpha_h(v) &= 0.128\exp(-(v+50)/18), \\
\beta_h(v) &= \frac{4}{1+\exp(-(v+27)/5)}, \\
\alpha_n(v) &= \frac{0.032(v+52)}{1-\exp(-(v+52)/5))}, \\
\beta_n(v) &= 0.5\exp(-(v+57)/40)\;.
\end{aligned}
$$

The parameter values of the E-cell model are $C = 1$ μF/cm^2, $g_{Na} = 100$ mS/cm^2, $g_K = 80$ mS/cm^2, $g_L = 0.1$ mS/cm^2, $v_{Na} = 50$ mV, $v_K = -100$ mV, and $v_L = -67$ mV.

We denote in general the number of E-cells in our network by N_e, and the number of I-cells by N_i. We adopt the synaptic model of ref. [2]. Each synapse is characterized by a synaptic gating variable s associated with the presynaptic neuron, with $0 \leq s \leq 1$. This variable obeys

$$\frac{ds}{dt} = H(v)\frac{1-s}{\tau_R} - \frac{s}{\tau_D}, \tag{S6}$$

where H denotes a smoothed Heaviside function:

$$H(v) = \frac{1+\tanh(v/4)}{2}, \tag{S7}$$

and τ_R and τ_D are the rise and decay time constants, respectively. To model the synaptic input from neuron j to neuron k, we add to the right-hand side of the equation governing the membrane potential v_k of neuron k a term of the form

$$g(j,k)s_j(t)(v_{rev} - v_k),$$

where $g(j,k)$ denotes the maximal conductance density associated with the synapse, s_j denotes the gating variable associated with neuron j, and v_{rev} denotes the synaptic reversal potential. For AMPA-receptor-mediated synapses, we use $\tau_R = 0.1$ ms, $\tau_D = 3$ ms, and $v_{rev} = 0$ mV; for GABA$_\text{A}$-receptor-mediated synapses, $\tau_R = 0.3$ ms, $\tau_D = 9$ ms, and $v_{rev} = -80$ mV. We also refer to the maximal conductance $g(j,k)$ as the "strength" of the synapse.

The strength of the synaptic connection from the j-th E-cell to the k-th I-cell is random, chosen prior to the beginning of the simulation and fixed from then on:

$$g_{ei}(j,k) = \begin{cases} \gamma_{ei} & \text{with probability } p_{ei}, \\ 0 & \text{with probability } 1 - p_{ei}, \end{cases}$$

where γ_{ei} and p_{ei} are independent of j and k, $\gamma_{ei} > 0$ and $0 < p_{ei} \leq 1$. We define

$$G_{ei}(k) = \sum_{j=1}^{N_e} g_{ei}(j,k)$$

to be the sum of all excitatory synaptic conductance densities impinging upon the k-th I-cell, and

$$\overline{G}_{ei} = \frac{1}{N_i} \sum_{k=1}^{N_i} G_{ei}(k).$$

Analogous formulas define g_{ie}, G_{ie}, $\overline{G}_{ie}$, g_{ii}, G_{ii}, $\overline{G}_{ii}$, g_{ee}, G_{ee}, and $\overline{G}_{ee}$. We do not include E-to-E-synapses throughout most of this paper (so usually $g_{ee}(j,k) = 0$ for all j and k), but see Supporting Information C.

The strength of the external input to the j-th E-cell is also random, chosen prior to the beginning of the simulation and fixed throughout:

$$I_{e,j} = (1 + r_e Z_j)\overline{I}_e, \tag{S8}$$

where $\overline{I}_e$ is independent of j, the Z_j are independent standard Gaussians, and $r_e \geq 0$. We say then that the heterogeneity in the external drives to the E-cells is $r_e \times 100\%$. For instance, when $r_e = 0.15$, we say that there is 15% heterogeneity in the drives to the E-cells. The strength of the external input to the k-th I-cell is defined by

$$I_{i,k} = \overline{I}_i + r_i U_k, \tag{S9}$$

where the U_k are independent random variables uniformly distributed in $[-1,1]$, and $r_i \geq 0$. We do not use a formula exactly analogous to Eq. (S8) for $I_{i,k}$ because drives to the I-cells will often be taken close to zero, and it can therefore be misleading to specify relative fluctuations of those drives.

In Fig. 3, $N_e = 80$ and $N_i = 20$. In panels A–I, $\overline{G}_{ie} = 0.3$ mS/cm^2 and $\overline{G}_{ii} = 0.05$ mS/cm^2. In panels A, D, and G, $\overline{G}_{ei} = 0.12$ mS/cm^2. In panels B, E, and H, $\overline{G}_{ei} = 0.08$ mS/cm^2. In panels C, F, and I, $\overline{G}_{ei} = 0.04$ mS/cm^2. In panels A–C, $p_{ei} = p_{ie} = p_{ii} = 1$. In panels D–F, $p_{ei} = 0.5$ and $p_{ie} = p_{ii} = 1$. In panels G–I, $p_{ei} = p_{ie} = p_{ii} = 0.5$. In all panels, $\overline{I}_e = 1.5$ μA/cm^2 and $\overline{I}_i = 0$ μA/cm^2. In panels A–F, external drives are homogeneous: $r_e = r_i = 0$. In panels G–I, they are heterogeneous: $r_e = 0.15$ and $r_i = 0.2$.

The parameters in panel J are the same as in panel I, except $\overline{I}_i = 0.4$ μA/cm^2 and $\overline{G}_{ii} = 0$ mS/cm^2.

Figure 4: The rhythmicity measure ρ associated with a network is defined as follows. We simulate the network starting at time $t = T_0 = -100$ ms and ending at time $t = T_1 = 1,000$ ms. To avoid initialization effects, we disregard the initial 100 ms, analyzing the results for t between 0 and $T_1 = 1,000$ ms only. We denote the average of the synaptic gating variables associated with the E-cells by s_E. Thus our simulation generates $s_E(t)$, $t = k\Delta t$, with $k = 0,1,2,...,T_1/\Delta t$. We write $M = T_1/\Delta t$. In the simulations of this paper, we always use $\Delta t = 0.02$ ms, and therefore $M = 50,000$. We expand s_E into a discrete Fourier series of period T_1:

$$s_E(t) = \sum_{\nu=-M/2}^{M/2-1} \hat{s}_E(\nu) e^{i\nu 2\pi t/T_1}, \quad t = k\Delta t, \quad k = 0,1,2,...,M-1.$$

Note that the period of the Fourier mode $e^{i\nu 2\pi t/T_1}$ equals $T_1/\nu = 1,000/\nu$ ms, and therefore its frequency is ν Hz. We define

$$\rho = \frac{\sqrt{\sum_{30\le|\nu|\le 50} |\hat{s}_E(\nu)|^2}}{\sqrt{\sum_{-M/2\le\nu<M/2} |\hat{s}_E(\nu)|^2}} \,. \tag{S10}$$

By Parseval's identity, the denominator of the right-hand side of Eq. (S10) equals, up to a factor of $\sqrt{\Delta t/T_1}$, the energy (i.e., discrete L^2-norm) of $s_E = s_E(t)$, $0 \le t < T_1$. Similarly, the numerator, up to the same factor, equals the energy of the component of s_E in the gamma range, defined for the purposes of this figure to be the 30–50 Hz range. (In the simulations of Fig. 4, there are no significant rhythms outside the 30–50 Hz range.) Thus ρ is the fraction of the energy of s_E that lies in the gamma range.

Figure 5: The E-cell is defined as described earlier in the supporting information about Fig. 3. The drive to the E-cell is $I_e = 1.6$ μA/cm^2, far above the spiking threshold. We assume that at time zero, the point (v,n,h) lies on the limit cycle, and $v = 0$ mV, $dv/dt > 0$. (These conditions determine (v,n,h) uniquely). We add to the right-hand side of the equation describing the evolution of the membrane potential v of the E-cell a term of the form

$$\begin{cases} G_{ie}e^{-(t-t_*)/\tau_i}(v_{inh}-v) & \text{if } t \ge t_*, \\ 0 & \text{if } t < t_*, \end{cases}$$

where $v_{inh} = -80$ mV denotes the reversal potential of inhibitory synapses, and $\tau_i = 9$ ms is the decay time constant of inhibition. We denote by $\hat{T}$ the time of the next spike, where "time of spike", as before, means the time at which v rises above 0 mV, i.e., $v = 0$ mV and $dv/dt > 0$. We define $\delta = \hat{T} - t^*$. Figure 5 shows δ as a function of t^* for $G_{ie} =$ (A) 0.24, (B) 0.12, (C) 0.06, and (D) 0 mS/cm^2.

Figure 6: This is a network simulation as in Fig. 3. In all panels of the figure, $\overline{G}_{ei} = 0.12$ mS/cm^2 and $\overline{G}_{ii} = 0.05$ mS/cm^2. In panel A, $\overline{G}_{ie} = 0.2$ mS/cm^2. In panel B, $\overline{G}_{ie} = 0.05$ mS/cm^2, and in panel C, $\overline{G}_{ie} = 0.02$ mS/cm^2. In all panels, $p_{ei} = p_{ie} = p_{ii} = 1$, $\overline{I}_e = 1.5$ μA/cm^2, $\overline{I}_i = 0$ μA/cm^2, $r_e = r_i = 0$.

Figure 7: Same as Fig. 6, except $p_{ei} = 0.5$, $r_e = 0.1$.

Figure 8: Panel A is a closeup of panel A of Fig. 7. In Panel B, the parameter values are those of Fig. 7 as well, except $\overline{G}_{ie}$ is ten times greater: $\overline{G}_{ie} = 2.0$ mS/cm^2.

Figure 9: The quantity ρ plotted in Fig. 9 is essentially that plotted in Fig. 4 (see Eq. (S10)), with one modification: We define the gamma range to be the range from 30 to 60 Hz here. The reason is that the rhythms become fairly fast as $\overline{G}_{ie}$ is weakened, making a wider definition of the gamma range more appropriate. Thus the definition of ρ used in

Fig. 9 is

$$\rho = \frac{\sqrt{\sum_{30 \le |\nu| \le 60} |\hat{s}_E(\nu)|^2}}{\sqrt{\sum_{-M/2 \le \nu < M/2} |\hat{s}_E(\nu)|^2}} . \tag{S11}$$

Figure 10. This is an E/I-network like those in the previous figures, but with $N_e = 20$ and $N_i = 1$. In all three panels of the figure, $\overline{G}_{ei} = 0.12$ mS/cm^2 and $\overline{G}_{ii} = 0.05$ mS/cm^2, $p_{ei} = p_{ie} = p_{ii} = 1$, the drive to the j-th E-cell is $(1.38 + 0.02j)$ μA/cm^2, and the drive to the I-cell is zero. In panel A, $\overline{G}_{ie} = 0.24$ mS/cm^2; in panel B, $\overline{G}_{ie} = 0.12$ mS/cm^2; in panel C, $\overline{G}_{ie} = 0.06$ mS/cm^2.

Figures 11 and 12: All information is in the figure captions.

Figure 13: Here all E-cells receive stochastically fluctuating input, and some receive constant input in addition. The stochastic component of the drive to an E-cell is modeled by an additional term on the right-hand side of the evolution equation for the membrane potential of the form $-0.05 s_{stoch}(t)\, v$. The gating variable s_{stoch} decays exponentially with time constant 3 ms during each time step. At the end of each time step, s_{stoch} jumps up to 1 with probability $\Delta t/(25 \text{ ms})$, where $\Delta t > 0$ denotes the duration of a time step. This simulates the arrival of external synaptic input pulses on an approximate Poisson schedule, at a mean frequency of 40 Hz. (Even though we measure time in ms, we measure frequencies in Hz.) Different cells in the network receive independent stochastic input streams.

The network in Fig. 13 is larger than in previous simulations: $N_e = 320$ and $N_i = 80$. All synapses are strong: $\overline{G}_{ie} = 0.4$ mS/cm^2, $\overline{G}_{ei} = 0.2$ mS/cm^2, $\overline{G}_{ii} = 0.1$ mS/cm^2. Synaptic connections are sparse and random: $p_{ei} = 0.5$, $p_{ie} = p_{ii} = 0.75$. All E-cells receive a homogeneous base tonic drive of strength 0.2. In addition, the first m E-cells receive heterogeneous additional tonic drive of strength 2, with $r_e = 0.2$, and $m = 250$ (A), $m = 150$ (B), $m = 50$ (C), and $m = 0$ (D). Drive to the I-cells is characterized by $\overline{I}_i = 0.4$ μA/cm^2 and $r_i = 0.2$.

The parameters in panel E are those of panel C, except $\overline{G}_{ei} = 0.6$ mS/cm^2.

Figure 14: Here we assign to each neuron a random location in the disk of radius 1 centered at the origin of the (x, y)-plane. (Distance is non-dimensionalized.) We choose these locations with a distribution that is approximately uniform, but avoids the clustering that is typical of truly uniformly distributed random points. The details are described below.

The locations of the E-cells and those of the I-cells are chosen independently. Suppose that N is the number of neuron locations to be chosen; $N = N_e$ for the E-cells, and $N = N_i$ for the I-cells. We choose a number $d \ge 1$ and a positive integer M, and cover the square $[-d, d] \times [-d, d]$ with M by M square cells of size $h \times h$, with $h = 2d/M$. Our choices of d and M will be stated and motivated shortly. In each of the cells of size $h \times h$, we choose one random location, with uniform distribution. This results in M^2 random points in the square $[-d, d] \times [-d, d]$. We then discard those points that lie outside the unit disk. The

total number of points retained is random. To compute its expectation, let

$$Q_l = \begin{cases} 1 & \text{if the point chosen in the } l\text{-th } h \times h\text{-cell belongs to the disk,} \\ 0 & \text{otherwise,} \end{cases}$$

$1 \le l \le M^2$. Let A_l denote the area of the intersection of the l-th $h \times h$-cell with the unit disk. Then

$$E(Q_l) = P(Q_l = 1) = \frac{A_l}{h^2}.$$

The expectation of the total number of points that fall into the unit disk is therefore

$$E\left(\sum_{l=1}^{M^2} Q_l\right) = \sum_{l=1}^{M^2} E(Q_l) = \sum_{l=1}^{M^2} \frac{A_l}{h^2} = \frac{\pi}{h^2} = \frac{\pi}{(2d/M)^2} = \frac{\pi}{4d^2} M^2.$$

We would like to choose M and d such that

$$\frac{\pi}{4d^2} M^2 = N,$$

i.e.,

$$M^2 = d^2 \frac{4N}{\pi}.$$

We therefore choose a positive number d, as small as possible but ≥ 1, such that $4d^2N/\pi$ is the square of an integer, then define $M = \sqrt{4d^2N/\pi}$.

The number of points in the unit disk generated in this way is random, with expected value N. To obtain exactly N locations, we simply repeat the procedure, with different initial seeds for the random number generator, until, by chance, the exact number of selected locations becomes N. (This never takes very long, and is not an important part of the cost of our simulations.)

Figures 15, 16, and 18 show results of simulations in which the neurons are placed in this way in the unit disk, and connection probabilities decay with distance. In these simulations, the E-cells in a smaller disk at the center of radius R are driven strongly; an example is indicated in yellow in Fig. 14.

Figure 15. There are $N_e = 320$ E-cells and $N_i = 80$ I-cells, with random spatial locations in a unit disk as described in the supporting information on Fig. 14. We let the probability (not the strength) of E-to-I-connections decay as follows:

$$g_{ei}(j,k) = \begin{cases} \gamma_{ei} & \text{with probability } e^{-(d_{jk}/\delta_{syn})^2}, \\ 0 & \text{otherwise,} \end{cases} \tag{S12}$$

where d_{jk} is the (non-dimensionalized) spatial distance between the j-th E-cell and the k-th I-cell, $\delta_{syn} > 0$ is the (non-dimensionalized) length scale characterizing the decay of the probability of synaptic connections, and $\gamma_{ei} > 0$ is the strength of an individual E-to-I-synapse. The I-to-E and I-to-I-synapses are described by analogous formulas, with the same decay length scale δ_{syn}.

The strengths of individual synapses are $\gamma_{ei} = 0.006$ mS/cm^2 and $\gamma_{ie} = \gamma_{ii} = 0.024$ mS/cm^2. The length scale δ_{syn} characterizing the decay of connection probabilities is taken to be 0.25. The E-cells in the disk centered around the origin with radius R receive constant heterogeneous drive with $\overline{I}_e = 1.5$ μA/cm^2, $r_e = 0.2$, with $R = 0.8$ (panel A), $R = 0.6$ (B), $R = 0.4$ (C), and $R = 0.2$ (D). The other E-cells receive no external drive ($\overline{I}_e = 0$ μA/cm^2). For all I-cells, $\overline{I}_i = 0.2$ μA/cm^2 with $r_i = 0$.

Figures 16 and 17. All information is in the figure captions.

Figure 18. Parameters in Fig. 18A are precisely as in Fig. 16A. In panels B and C of Fig. 18, the parameters are the same except for δ_{syn}, which is reduced to 0.20 in panels B and C, and the synaptic strengths, which are tripled in panel C: The strengths of individual synapses are $\gamma_{ei} = 0.009$ mS/cm^2 and $\gamma_{ie} = \gamma_{ii} = 0.036$ mS/cm^2 in panel C.

Numerics. All differential equations were solved using the explicit midpoint method with time step $\Delta t = 0.02$ ms.

C. Effect of E-to-E-synapses on PING. We have left out E-to-E synapses in the model networks used in this paper. Here we present numerical experiments showing that to first approximation, E-to-E-synapses simply add excitation to the E-cells, raising the PING frequency but not affecting strong PING in a qualitative way. The additional excitation can of course be crucial, as illustrated for instance by Spencer [6, Fig. 1]. However, in this paper, we provide the needed excitation of the E-cells via external drive.

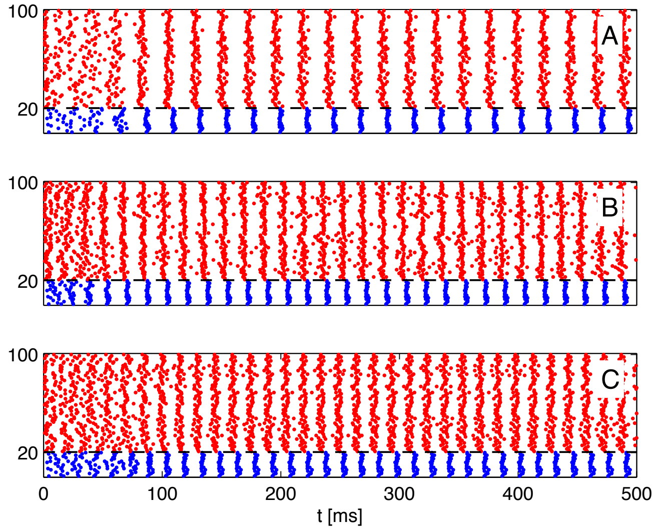

Figure S1: **Effects of E-to-E-synapses on PING rhythm.** Panel A: Same as panel A of Fig. 7. Panels B and C: E-to-E-synapses added. In both panels, $\tau_R = 0.1$ and $p_{ee} = 0.5$. Panel B: $\tau_D = 3$ ms, $\overline{G}_{ee} = 0.1$ mS/cm^2. Panel C: $\tau_D = 100$ ms, $\overline{G}_{ee} = 0.02$ mS/cm^2.

Figure S1A is identical with Fig. 7A. In panels B and C of Fig. S1, we have added rapidly (panel B) or slowly (panel C) decaying E-to-E-synapses; for the details, see the figure caption. The E-to-E-synapses raise the frequency from 44 Hz (panel A) to 60 Hz (panel B) or 68 Hz (panel C).

Figure S1 supports the assertion that the effect of E-to-E-synapses is somewhat akin to the effect of raising external drive to the E-cells. We think, however, that the role of E-to-E-synapses in PING rhythms is worth further study in the future. Both slow and fast E-to-E-synapses do affect *weak* PING: Not surprisingly, they can greatly raise the rate at which the E-cells participate in the rhythm.

D. Effect of heterogeneity on synchronization of I-cells by E-cells when excitation is weak: Analysis of a model problem. For the purposes of the following discussion, we model an I-cell as the integrate-and-fire neuron defined by

$$v' = -\frac{v}{\tau} + I \quad \text{for } v < 1, \tag{S13}$$

$$v(t+0) = 0 \quad \text{if} \quad v(t-0) = 1. \tag{S14}$$

When using integrate-and-fire neurons, we non-dimensionalize most physical variables. In particular, v denotes non-dimensionalized membrane potential, shifted and scaled in such a way that the re-set value is 0 and the spiking threshold is 1. However, since firing frequency is crucial to our study, and since we are accustomed to measuring it in Hz, we leave the time t dimensional, measuring it, as everywhere else, in ms. As a result, τ is also a time (also measured in ms), and I is a reciprocal time (measured in ms^{-1}). We assume that the neuron does not spike intrinsically: $\tau I < 1$. (Note that τI is non-dimensional.)

Suppose that at time $t = 0$, v is at its equilibrium:

$$v(0) = v_{eq} = \tau I,$$

and then an excitatory input pulse arrives, altering the sub-threshold equation as follows:

$$v' = -\frac{v}{\tau} + I + w\varphi(t) \tag{S15}$$

for $t \geq 0$, as long as $v < 1$, where $w > 0$ is, like I, a reciprocal time, $\varphi \geq 0$, and $\int_0^\infty \varphi(t)dt = 1$. (For simplicity, we model the excitatory pulse as current, not synaptic input. Note that due to the partial non-dimensionalization explained in the previous paragraph, I and w are not, dimensionally, currents here, but reciprocal times.) We are interested in how variations in I or w affect the time that it takes to reach the spiking threshold. We show that if the excitatory pulse can only just barely elicit a response, the dependence of the time to spike on the parameters I and w becomes great.

Note that

$$v(t) = \tau I e^{-t/\tau} + \int_0^t (I + w\varphi(u))\, e^{(u-t)/\tau} du$$

for $t \geq 0$, as long as $v < 1$. Let T_s denote the time at which v reaches the threshold 1, assuming that it ever does. Then

$$\tau I e^{-T_s/\tau} + \int_0^{T_s} (I + w\varphi(u))\, e^{(u-T_s)/\tau} du = 1. \tag{S16}$$

We note for later reference that this equation can be re-written as

$$\int_0^{T_s} \varphi(u) e^{(u-T_s)/\tau} du = \frac{1 - \tau e^{-T_s/\tau} I - T_s I}{w}. \tag{S17}$$

Note that T_s is a function of w and I. We are interested in understanding how sensitively T_s depends on w and I, i.e., in the effects of heterogeneity in w and I. We therefore analyze the derivatives $\partial T_s/\partial w$ and $\partial T_s/\partial I$.

Differentiating Eq. (S16) with respect to w, we find

$$-I e^{-T_s/\tau} \frac{\partial T_s}{\partial w} + (I + w\varphi(T_s)) \frac{\partial T_s}{\partial w} + \int_0^{T_s} \varphi(u) e^{(u-T_s)/\tau} du$$
$$- \frac{1}{\tau} \frac{\partial T_s}{\partial w} \int_0^{T_s} (I + w\varphi(u)) e^{(u-T_s)/\tau} du = 0. \tag{S18}$$

Using Eq. (S17), Eq. (S18) simplifies as follows:

$$-I e^{-T_s/\tau} \frac{\partial T_s}{\partial w} + (I + w\varphi(T_s)) \frac{\partial T_s}{\partial w} + \frac{1 - \tau e^{-T_s/\tau} I - T_s I}{w}$$
$$- \frac{1}{\tau} \frac{\partial T_s}{\partial w} T_s I - \frac{1}{\tau} \frac{\partial T_s}{\partial w} \left(1 - \tau e^{-T_s/\tau} I - T_s I\right) = 0. \tag{S19}$$

We solve this equation for $w \partial T_s / \partial w$:

$$w \frac{\partial T_s}{\partial w} = - \frac{1 - \tau e^{-T_s/\tau} I - T_s I}{I + w\varphi(T_s) - 1/\tau}. \tag{S20}$$

Eq. (S15), together with $v(T_s) = 1$, implies that the denominator of Eq. (S20) is $v'(T_s)$:

$$w \frac{\partial T_s}{\partial w} = \frac{1 - \tau e^{-T_s/\tau} I - T_s I}{v'(T_s)}. \tag{S21}$$

We now assume that I is near 0, so that the model I-cell is driven significantly below the spiking threshold. For $I = 0$, Eq. (S21) becomes

$$w \frac{\partial T_s}{\partial w} = \frac{1}{v'(T_s)}. \tag{S22}$$

We interpret Eq. (S22) as follows. The left-hand side is the change in T_s, namely ∂T_s, divided by the relative change in w, namely $\partial w / w$. When the input pulse is strong, the time

that v takes to rise from its resting value to the threshold value should be on the order of a few milliseconds at most, and $v'(T_s)$ should not be very much smaller than 1. On the other hand, when the input pulse is only just barely able to trigger a response in the I-cell, then $v'(T_s)$ is nearly zero; in this case, and only in this case, the right-hand side of Eq. (S21) is large.

Similarly, differentiating Eq. (S16) with respect to I, we find

$$\tau e^{-T_s/\tau} - I e^{-T_s/\tau}\frac{\partial T_s}{\partial I} + (I + w\varphi(T_s))\frac{\partial T_s}{\partial I} + \int_0^{T_s} e^{(u-T_s)/\tau} du$$
$$- \frac{1}{\tau}\frac{\partial T_s}{\partial I}\int_0^{T_s}(I + w\varphi(u))e^{(u-T_s)/\tau} du = 0. \tag{S23}$$

Using Eq. (S17), Eq. (S23) becomes

$$\tau e^{-T_s/\tau} - \frac{1}{\tau}\frac{\partial T_s}{\partial I} + (I + w\varphi(T_s))\frac{\partial T_s}{\partial I} + \int_0^{T_s} e^{(u-T_s)/\tau} du = 0.$$

Evaluating the integral in this equation yields

$$-\frac{1}{\tau}\frac{\partial T_s}{\partial I} + (I + w\varphi(T_s))\frac{\partial T_s}{\partial I} + \tau = 0,$$

or

$$\frac{\partial T_s}{\partial I} = -\frac{\tau}{-1/\tau + I + w\varphi(T_s)},$$

or

$$\frac{\partial T_s}{\partial I} = -\frac{\tau}{v'(T_s)}. \tag{S24}$$

Thus $\partial T_s/\partial I$ can get large only if $v'(T_s)$ gets small, and as discussed earlier, this means that the input pulse is only just barely strong enough to elicit a response in the I-cell.

Note that Eq. (S24) implies that T_s depends sensitively on I if $v'(T_s)$ is small provided that τ is not small as well. We argue in Supporting Information F that τ should not be chosen much smaller than 10, since otherwise the intrinsic period of the integrate-and-fire neuron would become too sensitively dependent on I.

In Eq. (S24), the left-hand side is the change in T_s divided by the absolute change in I, not the relative change in I. By contrast, in Eq. (S22), the left-hand side is the change in T_s divided by the relative change in w. It is not natural to consider the relative change in I here, since we are primarily interested in values of I near zero; by contrast, w, the strength of the excitatory pulse, is not assumed to be near zero, and it is therefore natural to consider relative, not absolute changes in w in Eq. (S22).

E. Effect of heterogeneity on synchronization of E-cells by I-cells when inhibition is strong: Analysis of a model problem. We give here an analysis for integrate-and-fire neurons showing that the effects of heterogeneity in the strength of inhibition and in the

external drive to the E-cells is largely independent of the strength of inhibition, as long as inhibition is strong enough to bring a homogeneous population to approximate synchrony with a single inhibitory pulse, and the external drive to the E-cells is strong enough to drive a gamma-frequency rhythm. For theta neurons and quadratic integrate-and-fire neurons, similar results can be found in [1] and [3].

We consider a single integrate-and-fire neuron defined by Eqs. (S13), (S14), but now think of it as an E-cell, and assume supra-threshold drive: $\tau I > 1$; we examine how a strong inhibitory pulse acts on such a model neuron, and study in particular the dependence of the response on external drive and strength of inhibition. If this dependence is weak, then heterogeneity effects will be weak when an inhibitory pulse synchronizes a heterogeneous population of E-cells.

Assume that at time $t = 0$, a synaptic pulse of inhibition sets in, decaying exponentially with time constant $\tau_i > 0$. For simplicity, we assume that the synaptic reversal potential is the same as the reset potential, namely 0. (Recall that when using the integrate-and-fire model, we always assume that the membrane potential is shifted and scaled so that the reset potential is 0 and the firing threshold is 1.) Thus the equations governing v are

$$v' = -\frac{v}{\tau} + I - g e^{-t/\tau_i} v, \qquad \text{(S25)}$$

$$v(0) = v_0, \qquad \text{(S26)}$$

with $0 \le v_0 \le 1$. (Here g, like I, is a reciprocal time, measured in ms^{-1}.) Let $T_P > 0$ be the time at which v reaches the spiking threshold 1. (Since $\tau I > 1$, there is such a time.) If $v_0 = 0$, one can think of T_P as the period of the strong PING rhythm in a highly idealized setting.

Even in this very simple model, T_P is a function of five variables: I, g, τ, τ_i, and v_0. To make a complete parameter study feasible, we fix $\tau_i = 10$ ms and $\tau = 10$ ms. The choice $\tau_i = 10$ ms is motivated by the fact that the decay time constant of GABA_A-receptor-mediated inhibition is about 10 ms. We argue in Supporting Information F that τ should not be chosen much smaller than 10 ms, since otherwise the intrinsic period of the integrate-and-fire neuron would become too sensitively dependent on I. We have, however, tried other values of τ, and found that our conclusions are not substantially different for larger or (somewhat) smaller values of τ.

Now T_P depends on only three variables: $T_P = T_P(I, g, v_0)$, $I > 0.1$, $g > 0$, $v_0 \le 1$. We will focus on values of g large enough to cause approximate synchronization of a population of model neurons governed by (S25) with different initial values of v_0. We will assume that $g > I - 1/\tau$. Then the right-hand side of Eq. (S25) is negative for $t = 0$ and $v = 1$, so the pulse of inhibition prevents spiking for some positive amount of time, regardless of v_0. Consequently "$T_P(I, g, 1)$", which we define to be the limit of $T_P(I, g, v_0)$ as v_0 tends to 1 from below, is positive.

Unfortunately, not even for this very simple model is there a simple analytic answer to the question "For given I, how large does g have to be for synchronization to be tight?" However, the question can easily be answered numerically. For a given I, we define g_I to

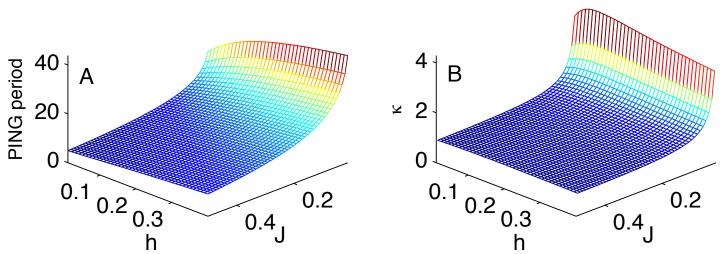

Figure S2: **PING period and its sensitivity as a function of drive and strength of inhibition.** A: PING period as a function of $J = I - 1/\tau$ and $h = g - g_I$. B: The "sensitivity measure" κ defined in Eq. (S27) as a function of J and h.

be the value of g for which $T_P(I, g, 1)$ differs from $T_P(I, g, 0)$ by exactly 10%:

$$\frac{T_P(I, g_I, 0) - T_P(I, g_I, 1)}{T_P(I, g_I, 0)} = 0.1.$$

(The choice of 10% is arbitrary here. Our results would be qualitatively very similar if we had chosen 5% or 50% here.) For $g \geq g_I$, reasonably tight synchronization is achieved by a single inhibitory pulse: All $T_P(I, g, v_0)$, $0 \leq v_0 \leq 1$, are within 10% of $T_P(I, g, 0)$. We now write $I = 1/\tau + J$, $J > 0$, and $g = g_I + h$, $h > 0$. Thus J is the amount of drive above the spiking threshold $1/\tau$, and h is the inhibitory conductance above the minimal conductance g_I needed for synchronization within 10% as a result of a single pulse. We plot $T_P(I, g, 0) = T_P(1/\tau + J, g_I + h, 0)$ as a function of J and h. This is shown in Fig. S2, panel A. In panel B of the same figure, we show the quantity

$$\kappa = \frac{I}{T_P(I, g, 0)} \frac{\partial T_P(I, g, 0)}{\partial I}, \tag{S27}$$

plotted as a function of J and h. Note that the definition of κ can be re-written (omitting, for brevity, the arguments $(I, g, 0)$) as

$$\kappa = \frac{\partial T_P / T_P}{\partial I / I}.$$

A relative change in I causes a κ times greater relative change in the "PING period" $T_P(I, g, 0)$. If κ is large, then even modest heterogeneity in I can have a large effect. Thus κ is a measure of sensitivity to perturbations in I; it is what is called a "condition number" in Numerical Analysis.

Note that κ and the expressions on the left-hand sides of Eqs. (S22) and (S24) are of a similar nature. However, in Eqs. (S22) and (S24), we considered absolute, not relative changes in T_s. In Eqs. (S22) and (S24), T_s is expected to be small, since it is the delay between the excitatory spike volley and the response of the inhibitory cells; therefore relative changes in T_s are less informative than absolute ones. Similarly, in Eq. (S24), we considered absolute, not relative changes in I because we thought of I as small. Here we think of neither T_P nor I as small, and therefore consider relative changes in both T_P and I.

Panel A of Fig. S2 indicates that the PING period never varies very rapidly with h (that is, with g). Thus as long as inhibition is strong enough to cause approximate synchronization in a single pulse ($h \geq 0$), heterogeneity in g should be expected to have moderate effects only. Panel B shows that the sensitivity measure κ is nearly independent of h, and becomes somewhat larger only when J gets small, i.e., when drive to the E-cell is near the firing threshold. (Panel A shows that in that case, the PING period is around 40 ms, corresponding to a 25 Hz oscillation, at the lower edge of the gamma range.)

F. The rhythm in the I-cells in Fig. 7C is an ING-rhythm. In Fig. 7C, there is a clear rhythm in the I-cells. We noted earlier that this is an ING-rhythm. Figure S3 confirms this point. Panel A of the figure shows a closeup of Fig. 7C (only spike times of 10 E-cells and 10 I-cells are shown), demonstrating that there is a rhythm in the I-cells. Panel B is the same as A, but with the I-to-I-synapses removed. Rhythmicity is clearly much reduced, although a remnant of rhythmic behavior still appears to be visible in the I-cells. We believe that this is an accidental finite-network-size effect. In fact, if we perform the same simulation in a twice larger network (Panel C), there appears to be yet less rhythmicity than in Panel B.

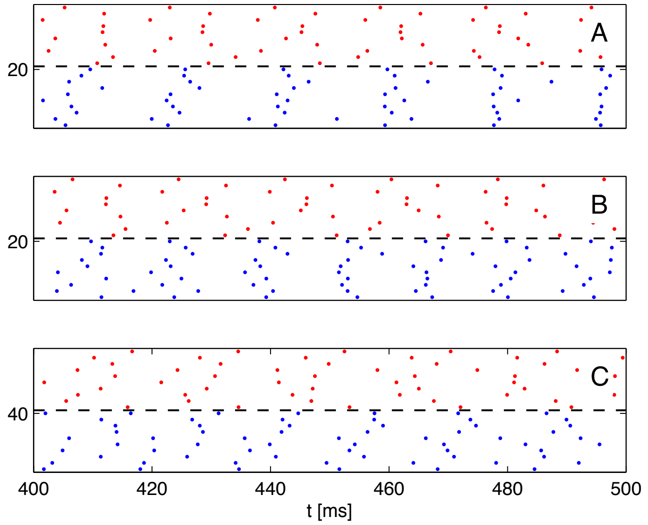

Figure S3: **The rhythm in Fig. 7C is ING.** A: Closeup of Fig. 7C (10 E-cells and 10 I-cells), demonstrating that there is a rhythm in the I-cells. B: Same as A, with the I-to-I-synapes removed. C: Same as B, with network size doubled. Although the simulated network was twice larger in panel C, the number of neurons for which spike times are displayed is the same (10 E- and 10 I-cells) in all three panels.

G. The "membrane time constant" of the integrate-and-fire neuron. For the analysis in Supporting Information D and E, it is essential that the parameter τ in the integrate-and-

fire model (S13), (S14) not be very small. One might think of τ as the "membrane time constant". However, τ also governs the nature of the dependence of the intrinsic firing frequency of the integrate-and-fire neuron on I. We will argue here that for $\tau \ll 10$ ms, that dependence becomes unreasonably sensitive.

The intrinsic period of the integrate-and-fire neuron defined by Eqs. (S13) and (S14) is

$$T = \tau \ln \frac{\tau I}{\tau I - 1}. \tag{S28}$$

With brief and straightforward calculations, Eq. (S28) implies

$$I = \frac{1}{\tau} \frac{e^{T/\tau}}{e^{T/\tau} - 1} \tag{S29}$$

and

$$\frac{dT}{dI} = -\frac{e^{T/\tau}}{I^2}.$$

To measure how sensitively T depends on I, it is natural to divide the relative change dT/T in T by the relative change dI/I in I, obtaining a "condition number", as in Supporting Information E:

$$\frac{dT/T}{dI/I} = \frac{I}{T}\frac{dT}{dI} = -\frac{e^{T/\tau}}{IT}. \tag{S30}$$

Using Eq. (S29) in Eq. (S30), we find:

$$\frac{dT/T}{dI/I} = -\frac{e^{T/\tau} - 1}{T/\tau}. \tag{S31}$$

The absolute value of the right-hand side of Eq. (S31) grows rapidly as T/τ increases, as shown in Fig. S4. For instance, if $T/\tau = 5$, a 1% increase in I causes a decrease in T by approximately 30%, according to this analysis. (The analysis is approximate because it is based on infinitesimal perturbations dI of I.)

In neuroscience, one is typically interested in neurons that spike with intrinsic periods T of several tens of milliseconds. If $\tau \ll 10$ ms, then the dependence of T on I becomes extremely sensitive for values of I that yield intrinsic periods of this order of magnitude.

Our analysis reflects a flaw of the integrate-and-fire model. In a more realistic neuronal model, the membrane time "constant" (which typically is not a constant, but varies as conductances vary) can be much smaller than 10 ms without there being any hyper-sensitivity issue.

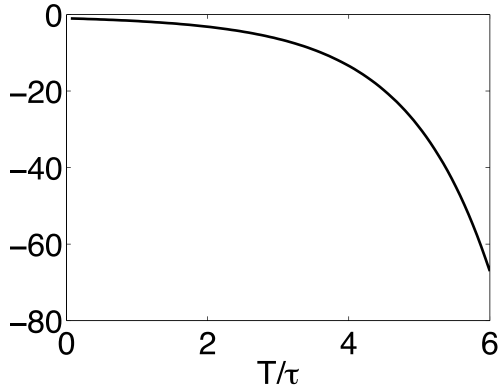

Figure S4: **Right-hand side of Eq. (S31) as a function of T/τ.**

References for Supporting Information

[1] C. Börgers and N. Kopell. Synchronization in networks of excitatory and inhibitory neurons with sparse, random connectivity. *Neural Computation*, 15(3):509–539, 2003.

[2] G. B. Ermentrout and N. Kopell. Fine structure of neural spiking and synchronization in the presence of conduction delay. *Proc. Natl. Acad. Sci. USA*, 95:1259–1264, 1998.

[3] N. Kopell and B. Ermentrout. Chemical and electrical synapses perform complementary roles in the synchronization of interneuronal networks. *Proc. Natl. Acad. Sci. USA*, 101(43):15482–15487, 2004.

[4] J. Y. Lin, M. Z. Lin, P. Steinbach, and R. Y. Tsien. Characterization of engineered channelrhodopsin variants with improved properties and kinetics. *Biophys. J.*, 96(5):1803–1814, 2009.

[5] M. Olufsen, M. Whittington, M. Camperi, and N. Kopell. New functions for the gamma rhythm: Population tuning and preprocessing for the beta rhythm. *J. Comput. Neurosci.*, 14:33–54, 2003.

[6] K. M. Spencer. The functional consequences of cortical circuit abnormalities on gamma oscillations in schizophrenia: insights from computational modeling. *Frontiers in Human Neuroscience*, 3:33, 2009.

[7] X.-J. Wang and G. Buzsáki. Gamma oscillation by synaptic inhibition in a hippocampal interneuronal network model. *J. Neurosci.*, 16:6402–6413, 1996.

[8] P. Welch. The use of fast Fourier transform for the estimation of power spectra: a method based on time averaging over short, modified periodograms. *Audio and Electroacoustics*, AU-15(2):70–73, 1967.
